# Extraction, purification, structural characterization, bioactivities, modifications and structure–activity relationship of polysaccharides from *Ophiopogon japonicus*: a review

**DOI:** 10.3389/fnut.2024.1484865

**Published:** 2024-11-13

**Authors:** Yiqiang Zhang, Bo Chen, Hua Zhang, Juan Zhang, Juan Xue

**Affiliations:** ^1^Department of Respiratory and Critical Medicine, Jingmen Hospital of Traditional Chinese Medicine, Jingmen, China; ^2^Department of Gastroenterology, The Second People’s Hospital of China Three Gorges University, Yichang, China; ^3^Department of Gastroenterology, The Second People’s Hospital of Yichang, Yichang, China; ^4^Department of Gastroenterology, Hubei Provincial Hospital of Integrated Chinese and Western Medicine, Wuhan, China; ^5^School of Clinical Medical, Hubei University of Chinese Medicine, Wuhan, China

**Keywords:** *Ophiopogon japonicus*, polysaccharides, extraction methods, structural characterization, biological activities, structure–activity relationship

## Abstract

*Ophiopogon japonicus* (Thunb.) Ker Gawl., is a traditional Chinese medicine (TCM) with a history of thousands of years. *O. japonicus* polysaccharides (OJPs), as one of the main active ingredient, are a kind of representative pharmacological bioactive macromolecules and mainly composed of Glc and Fru with molecular weight between 2.48 and 325 kDa. OJPs have a variety of biological activities, such as hypoglycemic, cardioprotective, immunomodulatory, improvement of obesity, and renal protective activity. In this review, the extraction, purification and structural characterization of OJPs were systematically reviewed, and its biological function, molecular mechanism and structure–activity relationship were analyzed. Additionally, this review summarized and found that structural modifications such as liposome modification, sulfation modification, and polyethylene glycol modification have improved the pharmacological activity and bioavailability of OJPs. Overall, this work will help to better understand OJPs and provide a theoretical review of their further development and utilization in the field of medicine and health food.

## 1 Introduction

**Figure 1:**
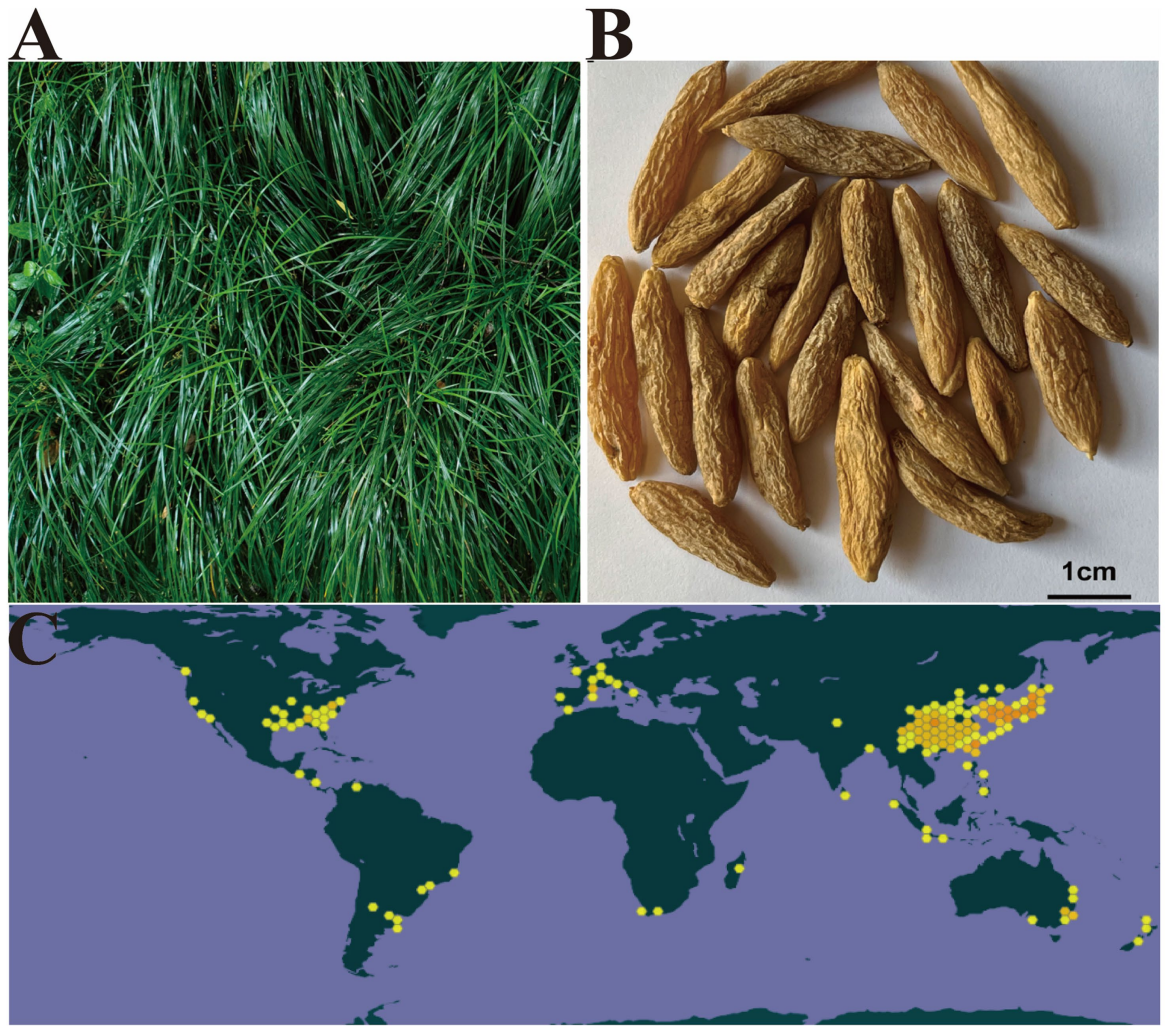
The herb of *Ophiopogon japonicus* **(A)** the dry root tuber of *O. japonicus* (Adapted from (2) and The Lancet with permission from Elsevier) **(B)**, and the distribution diagram worldwide of *O. japonicus* (Adapted from (2) and The Lancet with permission from Elsevier) **(C)** *Ophiopogon japonicus* via GBIF.org.

*Ophiopogon japonicus* (Thunb.) Ker Gawl., also called dwarf lilyturf or Maidong, its tuberous roots is a well-known herb that has been used extensively in traditional Chinese medicine (TCM) for thousands of years (1). *O. japonicus* is a perennial evergreen herb in the lily family and mainly distributed in East, South, and South-east Asia (including China, Japan, Korea and India), as shown in Figure 1. It is known that *O. japonicus* was first recorded in the Shen Nong’s Herbal Classic and has been regarded as yin-tonifying medicine in the field of TCM (3). According to the records of *O. japonicus* in the 2015 edition of Chinese Pharmacopoeia and Shen Nong’s Herbal Classic, *O. japonicus* acts on the heart, lung and stomach meridians, slightly cool, tastes sweet and light bitter (4, 5). In addition, *O. japonicus* has the function of nourishing Yin and promoting fluid, moistening lung and clearing heart, mainly used for the treatment of lung dryness and cough, hematemesis, lung carbuncle, thirst quenching, dry throat and dry mouth, constipation and other diseases. Modern pharmacological studies have confirmed that *O. japonicus* contains various chemical components, such as steroid saponins, flavonoids, polysaccharides, organic acids and phenols, which were shown to exhibit various pharmacological activities, including anti-inflammatory, anti-diabetes, anti-oxidation, anti-tumor, neuroprotective, antiviral, liver protective etc. (6–8).

Many plants, animals and fungi distributed in nature contain polysaccharides. Polysaccharides are important natural macromolecules widely distributed in nature, which are composed of more than 10 monosaccharides linked by glycosidic bonds (9). As one of the important active components, polysaccharides are widely involved in a variety of physiological regulation processes and have been paid more and more attention in recent years. It cannot be ignored that *O. japonicus* is also rich in polysaccharides. It is reported that *O. japonicus* polysaccharides (OJPs) have a variety of biological activities, including anti-diabetes, anti-inflammatory, kidney protection, hepatoprotective and immunomodulatory activity (10–12). The expression of these biological activities is closely related to the diversity of their structures. Therefore, in order to obtain polysaccharides with different biological activities, many studies have used different extraction methods, purification methods and chemical structure modification methods to obtain polysaccharides with different molecular structures.

In view of the great development potential of OJPs and in order to fully understand OJPs, this review systematically summarized the extraction, purification methods, structural characteristics and pharmacological activities of OJPs in recent years. Finally, the structure–activity relationship and structural modification of OJPs were discussed. It is hoped that this review can provide a meaningful reference for the development and application of OJPs.

## 2 Extraction and purification of OJPs

### 2.1 Extraction of OJPs

Extraction refers to the method of retrieving valuable elements from a substance through different mechanical or chemical procedures, like solvent processing, distillation, desiccation, or centrifugal force. The biological activity of polysaccharides depends on its structure, and the extraction method is an important factor affecting the structure of polysaccharides, so it is very important to choose appropriate extraction methods to extract polysaccharides. The extraction methods of OJPs include hot water, ultrasound-assisted, microwave-assisted and enzyme extraction. In Table 1, we make a comprehensive comparison of the commonly used extraction methods and list their advantages and disadvantages.

**Table 1:** Comparison of advantages and disadvantages of extraction methods from OJPs.

Polysaccharides extraction methods	Advantages	Disadvantages	Reference
Hot extraction	Simple operation, simple process and economic	High temperature affects polysaccharide content, long time, low extraction rate and large energy consumption	(13)
Ultrasonic-assisted extraction	High extraction rate, low energy consumption, short extraction time	Ultrasonic instruments are needed and low efficiency of large-scale extraction	(16)
Enzyme-assisted extraction	Mild conditions, convenient operation, environmental protection and high extraction rate	Specific enzymes are needed and the influencing factors are complex	(19, 20)
Microwave-assisted extraction	Low consumption of energy and solvent, a higher extraction rate and a shorter extraction time	High equipment requirements and a specific extractant is required	(24)

Polysaccharides are macromolecular compounds with polar properties, which are soluble in polar solvents, such as water and ethanol. Hot water extraction is a popular and widely used laboratory extraction method for extracting polysaccharides (13). In this process, *O. japonicus* was mixed with hot water, stirred and filtered, and then precipitated and centrifuged with ethanol to obtain crude polysaccharide OJPs. Distilled water at 90°C was extracted for 3 times, and 95% ethanol was precipitated and concentrated to obtain OJP with an average polysaccharide content of 98.50% (14). Xiong et al. (15) treated the broken tubers of *O. japonicus* with 85% ethanol to remove pigments, polyphenols and monosaccharides, then stirred at 90°C for 4 h. the extract was deproteinized with 5% trichloroacetic acid (TCA) at −20°C and 15 min, 4 times the volume of ethanol precipitated at 4°C, the yield of crude polysaccharide OJP was 30.36 ± 0.87%. Hot water extraction has the advantages of low cost and simple operation, but it also has some disadvantages, such as low purity, long extraction time, need a lot of water. Meanwhile, extraction temperature, extraction time, extraction times, ratio of material to liquid and other factors can affect the extraction rate of polysaccharides (13).

Ultrasonic-assisted extraction is an effective technique for OJP extraction. Under the action of ultrasonic cavitation, mechanical effect and thermal effect, the extraction efficiency of polysaccharides can be improved and the structure of polysaccharides can be modified to improve its biological activity. Ultrasonic-assisted extraction method solves the problems of high temperature and low extraction efficiency of traditional water extraction method and alcohol precipitation method, However, too long ultrasonic-assisted extraction time may destroy the structure of OJPs. Meanwhile, due to the limitations of instruments and equipment in the industrial field, ultrasonic extraction cannot be widely used (16). Under ultrasonic conditions of 80 W for 10 s, intermittent for 15 s, repeated 90 times, ethanol precipitation, dialysis, concentration and lyophilization to obtain OJP, the extraction rate of 11.87% (17).

The enzyme-assisted extraction conditions are mild and the cell wall is hydrolyzed by related enzymes, which is not easy to destroy the structure of polysaccharides and can improve the extraction rate of polysaccharides (18). However, this method has more strict requirements on extraction temperature, pH and other conditions. The corresponding enzymes were selected according to the properties of polysaccharides. The commonly used enzymes are pectinase, cellulase, trypsin, and starch glucosidase (19, 20). A variety of enzymes (21) can also be used in the combined method. The mixed solution of 1% amylase, 0.50% papain, 2% cellulase and 1.80 kg *O. japonicus* powder was mixed and stirred at 55°C for 1 h. After boiling water extraction and ethanol precipitation, the yield of OJP crude polysaccharide was 0.88% (22).

As a new extraction technology, microwave-assisted extraction enters plant glands, trichomes and vascular systems through microwave energy, which increases intracellular pressure and eventually leads to cell wall rupture and polysaccharide release. Chen et al. (23) studied the effects of microwave power, extraction time and liquid–solid ratio on the yield of polysaccharides by response surface methodology, and finally determined that the best extraction process of OJP was microwave power 300 tW, extraction time 15 min and liquid–solid ratio 40:1. The microwave-assisted extraction method has high extraction rate and short extraction time, but the instrument is expensive, so it is not suitable for large-scale production (24).

A single extraction method for OJPs extraction will inevitably have some limitations. In the future, other extraction technologies, such as supercritical fluid extraction, subcritical water extraction, ultrasound-microwave synergistic extraction and ultrasound-assisted enzyme extraction, can be used to coordinate the extraction of polysaccharides and improve the extraction rate of polysaccharides (25). However, there is no report on which new extraction method can be used for industrial-scale OJPs extraction. It is well known that different extraction methods directly affect the chemical composition, structure and biological activity of polysaccharides *in vitro* and *in vivo*. Therefore, it is necessary to further study the relationship between extraction methods and the structure and biological activity of polysaccharides.

### 2.2 Purification of OJPs

After extraction, crude polysaccharides contain a large number of impurities, including proteins, pigments and other substances, which should be further isolated and purified to obtain higher purity polysaccharides. Ethanol precipitation is a simple and widely used method for the separation of OJPs. Polysaccharides are polyhydroxyaldehydes or ketones that are soluble in water. The addition of ethanol to the aqueous solution of polysaccharides can destroy the hydrogen bond in the aqueous solution of polysaccharides, thus reducing the solubility of polysaccharides in water and making the polysaccharides precipitate. Chen et al. (26) obtained OJP from *O. japonicus* by precipitating the crude extract with 95% EtOH (1:4, v/w). Zhang et al. (27) were precipitated with 80% ethanol and centrifuged to obtain OJP. In most studies, ethanol precipitation was used to obtain OJPs. However, the polysaccharides obtained by ethanol precipitation are not pure and are easy to wrap some small molecules and need to be further purified.

After ethanol precipitation, crude polysaccharides are mixed with protein, pigment and other impurities. Sevag method is usually used to remove protein from polysaccharide solution. The Sevag method is based on the denaturation of proteins in chloroform and other organic solvents. The polysaccharide extract was mixed with Sevag reagent [chloroform: n-butanol = 4:1] according to a certain proportion, shaken well, and the supernatant obtained by centrifugation was the polysaccharide solution for partial protein removal (11). The advantage of Sevag method is that the condition is mild and it will not cause the denaturation of polysaccharides, but the protein removal rate is not high, and it often needs multiple operations to achieve satisfactory results. The principle of TCA method is that it forms irreversible precipitation with protein under the action of organic acid, which has good deproteinization effect and high efficiency (28). Xiong et al. (15) used this method to remove protein and purify OJPs. However, TCA method is easy to destroy the original structure of polysaccharides and hydrolyze some polysaccharides. As proteins, papain and cellulase are usually used to hydrolyze proteins, so they can be used to remove proteins. There are many factors affecting the enzymatic hydrolysis process, and the order is enzyme dosage > pH > enzymatic hydrolysis time > enzymatic hydrolysis temperature (29). 3% papain was used to hydrolyze proteins in crude polysaccharides, and the yield of OJP was 55.20 ± 1.86% (30). The pigments in polysaccharides affect the color and purity of OJPs and need to be decolorized. The commonly used methods are activated carbon decolorization, macroporous resin adsorption decolorization and H_2_O_2_ method (31, 32). The macroporous adsorption resin has a porous frame structure, which can adsorb pigments through the specific surface area and pore size of the mesh to achieve the purpose of decolorization. Wang et al. (33) used D101 macroporous resin to remove pigments from OJP. Another study found that 2% activated carbon had the best decolorization effect at 50°C for 30 min (34).

Column chromatography is the most widely used method to purify OJPs. The main column chromatography methods are ion exchange column chromatography and gel filtration column chromatography. The commonly used ion exchange column filler, such as DEAE-52 cellulose column, is a kind of anion exchanger. The principle of adsorbing acidic polysaccharides instead of neutral sugars was used to separate neutral sugars and acidic sugars. In addition, the ion exchange column also has certain functions of decolorization and impurity removal. The commonly used gel column chromatography fillers are Superdex and Sephadex. Sephadex G series separates polysaccharides with different molecular weights according to the size of cross-linking. The greater the cross-linking is, the tighter the mesh structure is, so it is suitable for the separation of small molecular polysaccharides. The crude polysaccharides were deproteinized and dissolved in distilled water by Sevag method, then applied to DEAE-52 cellulose column, eluted, concentrated, dialyzed and lyophilized by increasing the concentration of NaCl (0.01 m ~ 1.0 m). Finally, the sample OJP2 was further purified by Sephadex G-150 (11). Zhang et al. (27) concentrated the deproteinized polysaccharide solution on a DEAE cellulose-52 column, eluted with distilled water, and collected the eluent. Wang et al. (17) used DEAE-52 cellulose column and Sephadex G-150 gel column to separate *O. japonicus* polysaccharide (POJ-U1a) with a molecular weight of 4.02 kDa. Lin et al. (35) extracted crude polysaccharides by water extraction and alcohol precipitation, and then purified OJP by DEAE Sepharose Fast Flow and Sephadex G-25 chromatographic columns.

In summary, the purification process of polysaccharides is divided into three steps. First of all, the crude polysaccharides were ethanol precipitation, deproteinized and decolorized. Then, the polysaccharides were fractionated by column chromatography, including ion exchange chromatography, gel permeation chromatography and affinity chromatography. Finally, uniform pure polysaccharides were obtained by concentration, dialysis, freeze-drying and other steps. Many methods can be used to purify polysaccharides, but there is no standard purification method at present. In addition, the current pure methods are mostly used in laboratories and are difficult to use in industry. Therefore, it is necessary to develop a low-cost, green, sustainable and efficient polysaccharide purification technology to obtain high-quality OJPs on an industrial scale. The characteristics of OJPs extraction and purification, including extraction methods, time, temperature, solid–liquid ratio, total yield, and purification techniques, are summarized in Table 2.

**Table 2:** A summary of OJPs extraction and purification methods.

Part	Extraction							Purification			References
	Polysaccharide fraction	Method of extraction	Liquid/solid ratio	Time (min)	Temperature °C	Power (w)	Extraction rate (%)	Polysaccharide fraction	Purification methods	Yield (%)	
*O. japonicus* root	–	Hot water extraction	–	180	90	–	–	OJP2	DEAE-52 cellulose column, Sephadex G-150	–	(11)
*O. japonicus* root	OJP1	Hot water extraction		180	90	–	–	OJP1	DEAE-52 cellulose column, Sephadex G-100	–	(14)
*O. japonicus* tuber	–	Hot water extraction			90	–	30.36 ± 0.87	OJP1	DEAE-Sepharose fast flow column, Sepharose 6 Fast Flow column	–	(15)
*O. japonicus* root	POJ-U1	Ultrasonic extraction		15	–	80	11.87	POJ-U1a	DEAE-52 cellulose column, Sephadex G-150 gel column	1.25	(17)
*O. japonicus* root	ROH	Enzyme-assisted extraction			55		0.88	ROHO5	DEAE Sepharose™ Fast Flow column	16.40	(22)
*O. japonicus* root	OJZ1	Microwave-assisted extraction	40:1	15		300	–	OJZP1	–	7.30	(23)
*O. japonicus* root	OJC1	Microwave-assisted extraction	40:1	15		300	–	OJCP1	–	12.80	(23)
*O. japonicus* root	LM1	Microwave-assisted extraction	40:1	15		300	–	LMP1	–	14.50	(23)
*O. japonicus* root	LS1	Microwave-assisted extraction	40:1	15		300	–	LSP1	–	14.60	(23)
*O. japonicus* root	–	Hot water extraction		180	90	–	–	OJP1	DEAE-52 cellulose column, Sephadex G-100	–	(26)
*O. japonicus* root	–	Hot water extraction	–	30	100	–	–	OJP	DEAE-52 cellulose column	55.20 ± 1.86	(30)

## 3 Structural characterization

**Figure 2:**
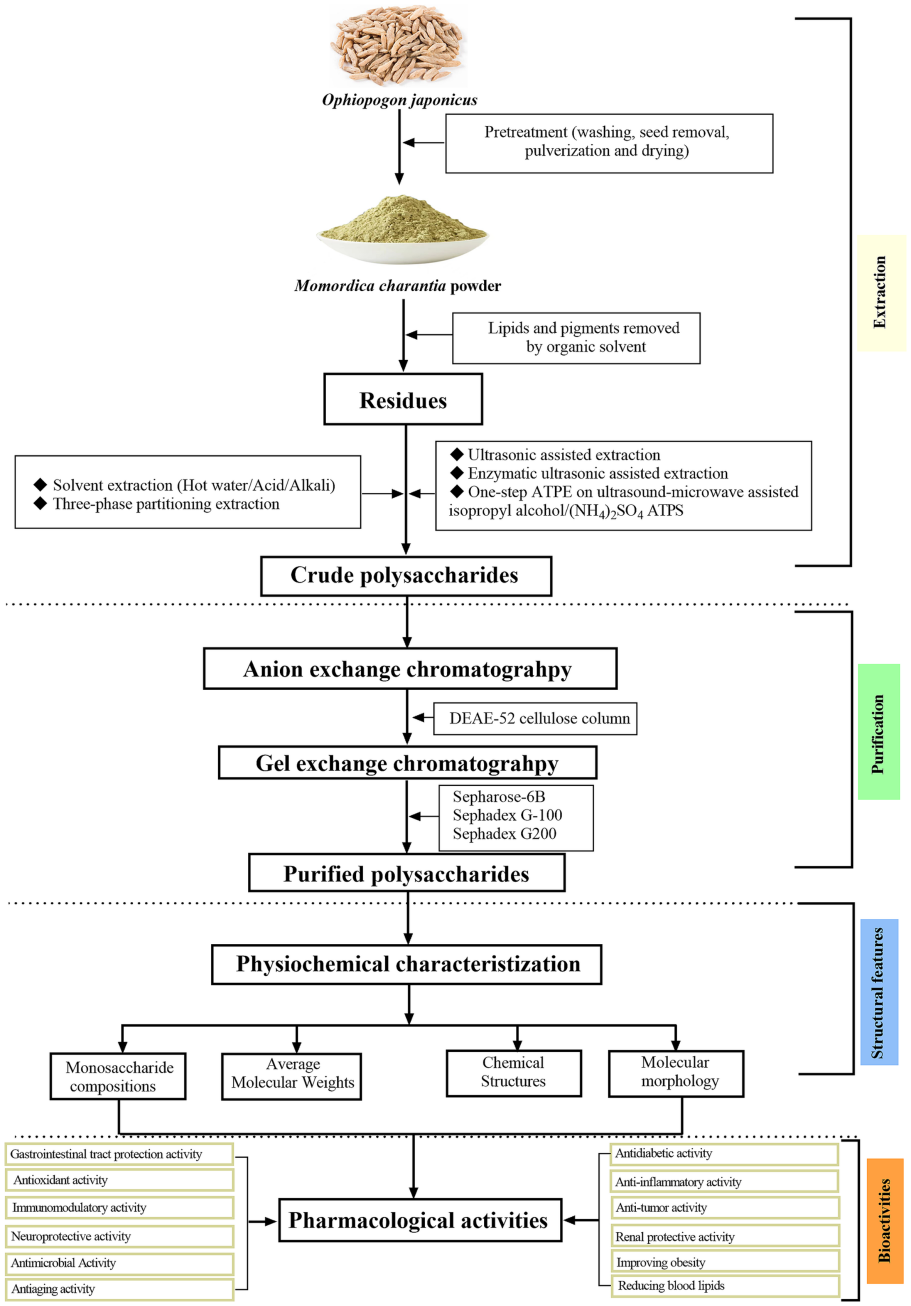
Schematic diagram of extraction, purification, structural features, and biological activities of polysaccharides isolated from *O. japonicus*.

The OJPs has a complex and diverse structure, which may be caused by different raw materials, extraction methods, purification methods and modification techniques. Due to the structural differences, OJPs has different physical and chemical properties and rich biological activities. The structural characteristics of OJPs were mainly determined by high performance liquid chromatography (HPLC), Fourier transform infrared spectroscopy (FTIR), gas chromatography (GC), nuclear magnetic resonance (NMR) and gas chromatography–mass spectrometry (GC–MS). These methods can be used to reveal the composition and structural characteristics of OJPs, so as to provide support for the biological activity and application of OJPs. Table 3 summarizes the information about OJPs, including name, molecular weight, monosaccharide composition, chemical structure, biological activity, structural characterization method and related references. The extraction, purification, structural features, and biological activities of OJPs are shown in Figure 2.

**Table 3:** The polysaccharides isolated from *Ophiopogon japonicus*.

No.	Compound name	Monosaccharide compositions	Molecular weight (kDa)	Possible structures	Bioactive activities	Structural characterization method	Reference
1	OJP2	Rha, Ara, Xyl, Glu, Gal = 0.50: 5: 4: 1: 10	88.10	2,5-Me-Xyl (1,3-linked Xyl), 2,3,4-Me-Gal (1,6-linked Gal) and part of Ara were major components of the backbone structure, part of Ara and Gal were distributed in branches, and residues of branches terminated with Rha, Glc and Gal	Antioxidant activity	HPGPC, GC–MS, GC, IR	(11)
2	OJP1	Ara, Glu, Gal =1: 16: 8	35.20		Hypoglycemic, hepatoprotective and renal protective activity	GC, HPGPC	(14)
3	OJP1	Man, Glu	2.74	Contained a small quantity of sulfate group and uronic acid	Antioxidant and immunoregulatory activity	GC, IR	(15)
4	OJP2	Rha, Xyl, Ara, Glu	124.30	Contained a small quantity of sulfate group and uronic acid	Antioxidant and immunoregulatory activity	GC, IR	(15)
5	OJP3	Ara, Glu	324.65	Contained a small quantity of sulfate group and uronic acid	Antioxidant and immunoregulatory activity	GC, IR	(15)
6	OJP4	Rha, Glu	6.75	Contained a small quantity of sulfate group and uronic acid	Antioxidant and immunoregulatory activity	GC, IR	(15)
7	POJ-U1a	–	4.02	The backbone of POJ-U1a was 1,6-α-d-glucopyranose and 1,3,6-α-d-glucofuranose in the molar ratio of 7:3, while the branched chains were mainly composed of 1,3-α-d-glucopyranose and 1-α-d-glucopyranose in the molar ratio of 1:3	Antioxidant activity	IR, ^13^C NMR, GC–MS	(17)
8	ROH05	Gal	16.70	1, 4-linked β-d-Galp and 1, 4, 6-linked β-d-Galp and branching chain containing T-linked β-d-Galp attached at C-6 position of 1, 4, 6-linked β-d-Galp		^13^C NMR, GC, GC–MS	(22)
9	OJZP1	Fru: Glc: Ara: Man: Gal: Xyl = 87.73: 5.57: 1.00: 0.94: 0.61: 0.31	6	–	Antioxidant and immunoregulatory activity	HPSEC-MALLS-RID	(23)
10	OJCP1	Fru: Glc: Ara: Man: Gal: Xyl = 164.22: 10.83: 1.00: 1.09: 0.87: 0.32	12	–	Antioxidant and immunoregulatory activity	HPSEC-MALLS-RID	(23)
11	LMP1	Fru: Glc: Ara: Man: Gal: Xyl = 161.76: 14.41: 1.00: 0.35: 0.58: 0.21	6.80	–	Antioxidant and immunoregulatory activity	HPSEC-MALLS-RID	(23)
12	LSP1	Fru: Glc: Ara: Man: Gal: Xyl = 236.06: 17.31: 1.00: 0.89: 0.64: 0.35	17	–	Antioxidant and immunoregulatory activity	HPSEC-MALLS-RID	(23)
13	OJP	Fru, Glu = 29: 1	4.93	The backbones were formed by Fruf-(2→, 2 → 2)-Fruf-(6→, →6)-Glcp-(1 → and → 1, 2)-Fruf-(6→)	Anti-diabetic activity	NMR, FT-IR, GC–MS	(30)
14	OJP	Fru, Glu = 17: 1	2.48		Promote ginsenoside metabolism		(38)
15	OGP	Glu	10.70		Immunoregulatory activity	HPGPC	(64)

Glc, glucose; Gal, galactose; Man, mannose; Ara, arabinose; Xyl, xylose; Rha, rhamnose; Glu, glucose; Fru, fructose; FT-IR, Fourier transform infrared spectroscopy; NMR, nuclear magnetic resonance; HPGPC, high-performance gel permeation chromatography; GC, gas chromatography; GC–MS, gas chromatography–mass spectrometry; HPSEC-MALLS-RID, high performance size exclusion chromatography coupled with multi-angle laser light scattering and refractive index detector; IR, infrared spectroscopy.

### 3.1 Monosaccharide compositions

Monosaccharide composition analysis is an important step in the structure analysis of polysaccharides. Monosaccharide composition analysis is generally carried out complete acid hydrolysis of polysaccharides, so that the glycosidic bonds of polysaccharides are completely broken. After hydrolysis, neutralization, filtration, derivatization and other treatments are required, and then GC, GC–MS and high performance anion exchange chromatography-pulsed amperometric detector (HPAEC-PAD) are used to determine (36, 37). The monosaccharides and molar ratio of OJPs obtained from different raw materials or different extraction processes are different, but OJPs mainly contains fructose (Fru) and glucose (Glc), a small amount of arabinose (Ara), mannose (Man), galactose (Gal) and xylose (Xyl). The POJ-U1a obtained by ultrasonic method was only composed of Glc by GC analysis (17). In another study, OJP was obtained by water extraction and alcohol precipitation and Sephadex G-25 column purification, structural analysis showed that OJP was composed of Fru and Glc at 17:1 (38). The OJP1, GC analysis of OJP1 was mainly composed of Ara, Glu and Gal, with a relative molar ratio of 1:16:8 by DEAE-52 cellulose and Sephadex G-100 purification (14). OJPs from different sources of *O. japonicus* were also extracted by microwave, and the monosaccharide composition of polysaccharides was determined by HPAEC-PAD. The results showed that the molar ratios of Fru to Glc in polysaccharides of Zhe MaiDong and Chuan MaiDong were about 15:1 and 14:1, respectively, indicating that the monosaccharide composition of polysaccharides from different sources was the same, but the content was not different (23). Detailed data on the composition of OJPs monosaccharides are shown in Table 3.

### 3.2 Molecular weights

The molecular weights of polysaccharides is one of the important factors affecting the expression of its biological activity. The accurate determination of the molecular weights of polysaccharides is the basis for analyzing the structure of polysaccharides. At present, the main methods to determine the molecular weights of polysaccharides are HPLC (39), high performance gel permeation chromatography (HPGPC) (40), high performance size exclusion chromatography coupled with multi-angle laser light scattering and refractive index detector (HPSEC-MALLS-RID) (41). It is worth noting that HPLC and HPGPC is the most commonly used method for determining the molecular weights of polysaccharides, which has the advantages of simple operation and high accuracy. HPSEC-MALLS-RID is a more powerful tool than HPGPC and HPLC, which can be used to determine molecular weight (Mw) of polysaccharides (42). Most of the Mw of OJPs obtained from different raw materials and extraction and purification methods range from 2.48 to 325 kDa, as shown in Table 3.

### 3.3 Chemical structure

Glycosidic bond is a special chemical bond between glycoside and glycoside or between glycosyl and glycosyl (43). The order or position of glycosyl groups affects the properties of polysaccharides (44). To obtain the primary structural repeat units of polysaccharides, a combination of periodate oxidation, smith degradation, methylation analysis, GC–MS, FT-IR, and NMR spectroscopy is required. A purified polysaccharide (MDG-1) was obtained by ultrafiltration, DEAE Sepharose FF and Sephadex G-25 column chromatography and contained a backbone composed of Fru*f* (2 → 1) and a branch of Fruf (2 → 6) Fruf (2→) per average 2.8 of main chain residues, it also contains trace of *α*-D-Glc (45). Wang et al. (17) analyzed the structure of POJ-U1a by GC, smith degradation, methylation analysis, GC–MS and NMR. It was found that POJ-U1a was an α-configuration polysaccharide with a highly branched structure, and consisted of pyranoside and funanside. The backbone of POJ-U1a was 1,6-α-d-glucopyranose and 1,3,6-α-d-glucofuranose in the molar ratio of 7:3, while the branched chains were mainly composed of 1,3-α-d-glucopyranose and 1-α-d-glucopyranose in the molar ratio of 1:3. A novel polysaccharide (OJP) with an hypoglycemic effect was isolated and purified, and the main chain of OJP was composed of Fruf-(2→, 2 → 2)-Fruf-(6→, →6)-Glc*p*-(1→ and →1,2)-Fruf-(6→ with a molar ratio of 6.80:15.80:1.00:5.80) (30). In addition, the results of GC–MS analysis showed that the main components of the OJP1 backbone structure were 2,3,4-Me-Glc (1,6-linked Glc), 2,3,6-Me-Glc (1,4-linked Glc), and 2,3-Me-Glc (1,4,6-linked Glc), part of Glc and Gal were distributed in branches, and residues of branches terminated with Ara, Glc, and Gal (26). A water-soluble polysaccharide (OJP2), was precipitated with 95% ethanol and purified by DEAE-52 cellulose anion-exchange and Sephadex G-100 gel filtration chromatography, the GC–MS results indicated that 2,5-Me-Xyl (1,3-linked Xyl), 2,3,4-Me-Gal (1,6-linked Gal) and part of Ara were major components of the backbone structure, part of Ara and Gal were distributed in branches, and residues of branches terminated with rhamnose (Rha), Glc and Gal (11).

**Figure 3:**
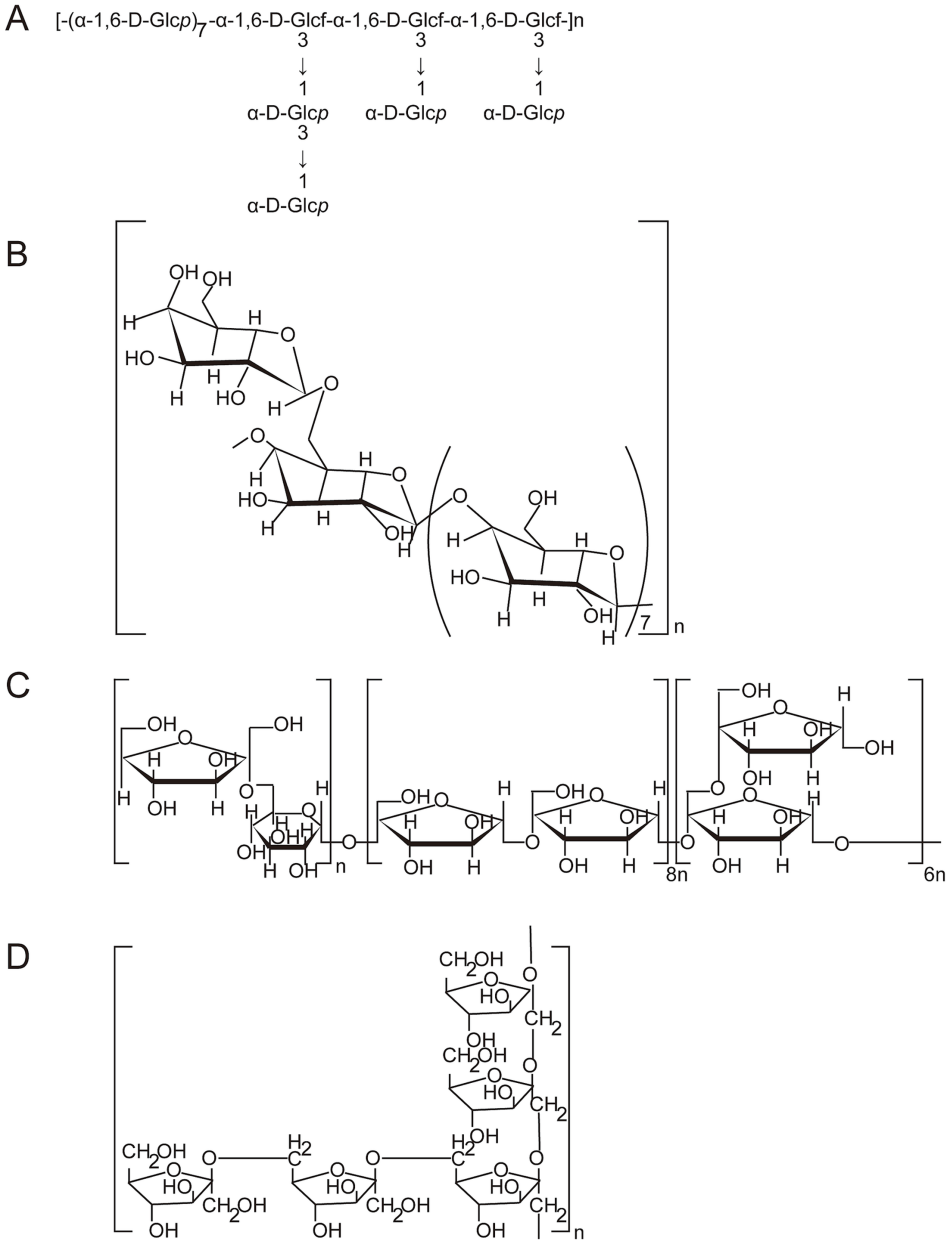
The possible structural features of OJPs. **(A)** The structural features of POJ-U1a (Reprinted from (17) and The Lancet, with permission from Elsevier). **(B)** The structural features of ROH05 (Reprinted from (22) and The Lancet, with permission from Elsevier). **(C)** The structural features of OJP (Reprinted from (30) and The Lancet with permission from Elsevier). **(D)** The structural features of ROP (Reprinted from (103) and The Lancet with permission from Elsevier).

From the above analysis, it can be seen that the OJPs obtained from different raw materials and separation and purification processes have structural diversity, which lays a solid structural foundation for the diversity of its biological activity. As shown in Figure 3, some of the structure of OJPs is shown. Future research should give priority to a more comprehensive understanding of primary and high-level structures and the relationship between structures and activities. In addition, the development of advanced and efficient technologies is essential for elucidating the chemical structures or functional groups that play a pharmacological role.

## 4 Biological activities of OJPs

The polysaccharides extracted from *O. japonicus* have been proved to have certain biological activities and are beneficial to human health. At present, a large number of studies have shown that OJPs has many biological activities, such as hypoglycemic, cardioprotective, immunomodulatory, improvement of obesity, hepatoprotective, hypolipidemia, anti-oxidation, anti-tumor, anti-inflammation, renal protective, and other biological activities.

### 4.1 Hypoglycemic activity

Diabetes mellitus is a series of metabolic disorders of protein, fat and electrolyte caused by insufficient absolute or relative insulin secretion and reduced sensitivity of target tissue cells to insulin (46). Hyperglycemia is the main feature of diabetes, which can lead to a variety of diseases, such as diabetic nephropathy, cardiovascular disease and retinopathy (47, 48). Research have shown that more than 100 million people in China have been diagnosed with diabetes, so finding and developing safe and effective hypoglycemic drugs plays a positive role (49).

**Figure 4:**
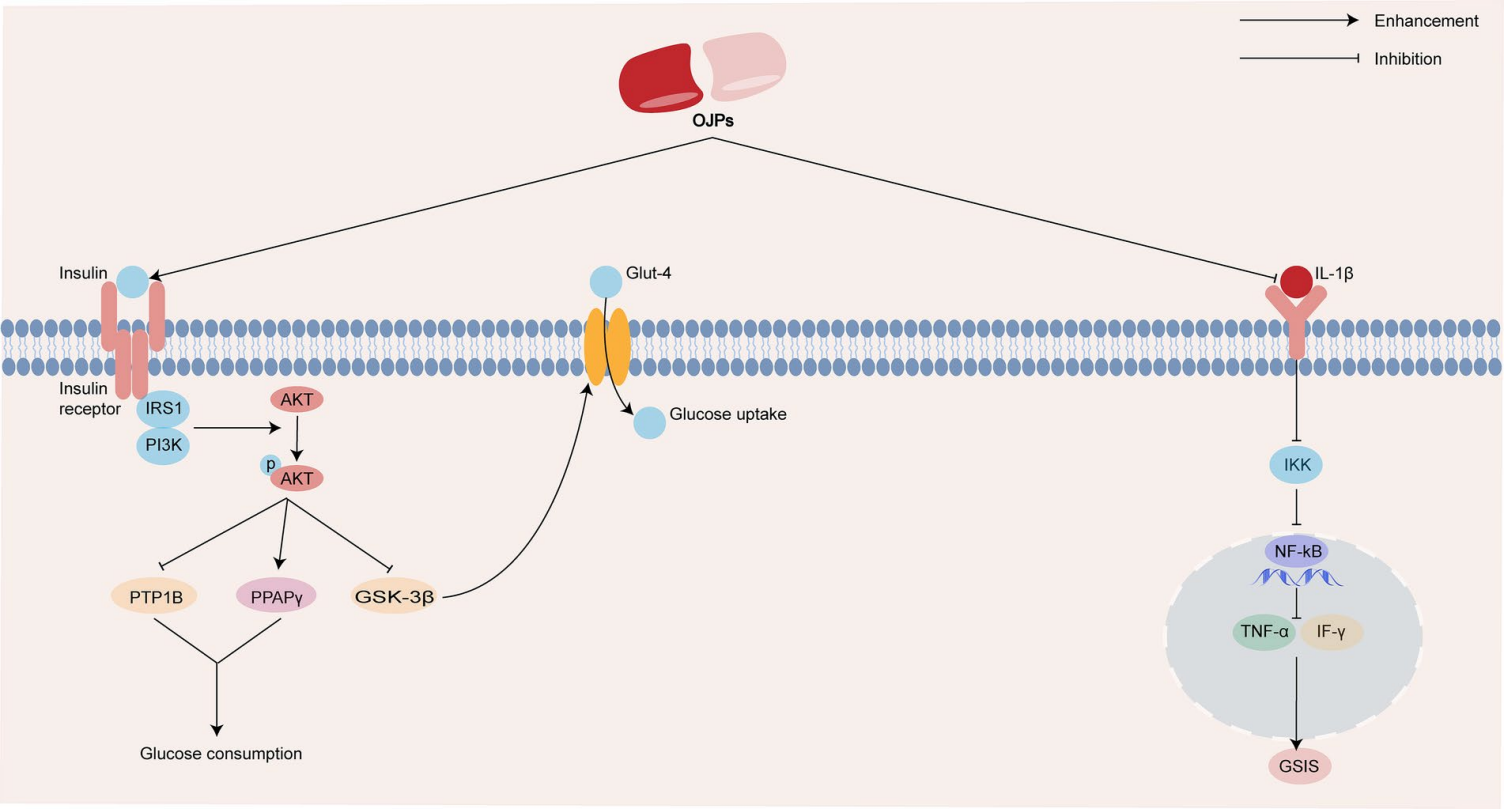
Schematic representation of the possible mechanism of antidiabetic activity of OJPs.

Studies have shown that OJPs have a positive therapeutic effect on diabetes. In the insulin resistance (IR)-induced HepG2 cell model, OJP up-regulated the mRNA and protein expressions of phosphoinositide 3-kinase (PI3K), protein kinase B (Akt), insulin receptor (InsR) and peroxisome proliferators-activated receptors *γ* (PPARγ) in IR HepG2 cells and decreased the mRNA and protein levels of protein tyrosine phosphatase 1B (PTP1B). This indicates that OJP improve the glucose uptake capacity of HepG2 cells by regulating the PI3K/Akt signaling pathway, thus playing a hypoglycemic role (30). Wang et al. (50) studied the hypoglycemic activity of OJP in diabetic KKAy mice. After the establishment of the model, the mice were fed with OJP for 75 and 300 mg/kg/days, respectively, for 8 weeks, the results showed that OJP could reduce blood glucose and improve insulin resistance in mice. In a follow-up study, OJP down-regulated the expression of PI3K/p85, AKT, InsR, insulin receptor submatter-1 (IRS-1) and glucose transporter type 4 (Glut-4), down-regulated the expression of glycogen synthase kinase 3β (GSK-3β), and played a hypoglycemic effect through InsR/InsR-1/PI3K/Akt/GSK-3/Glut-4 signaling pathway. Adiponectin is an endogenous protein secreted by mature adipocytes and plays an important role in the regulation of glucose metabolism and insulin sensitivity (51). As one of the types of diabetes mellitus, gestational diabetes may lead to abnormal embryo development, abortion and fetal deformity. Adiponectin levels in rats with gestational diabetes mellitus were significantly lower than those in normal rats, blood glucose and insulin levels after OJP intervention were significantly lower than those in the gestational diabetes mellitus model group, and adiponectin levels in serum, adipose tissue and placenta of rats with OJP group were significantly increased (52). Xu et al. (53) also found that oral OJP of 300 mg/kg significantly improved the oral glucose tolerance of diabetic mice, and reduced insulin level and triglyceride content. Interleukin-1*β* (IL-1β) can activate nuclear factor-κB (NF-κB) signal pathway and cause islet β cell dysfunction, which leads to hyperglycemia. *In vivo*, it was found that Maidong polysaccharides extract (MPE) protect pancreatic β cells, further cell experiments showed that MPE could reduce the gene expression of IL-1β, NF-κB, tumor necrosis factor-*α* (TNF-α) and interferon-*γ* (IFN-γ), thus reduce pancreatic inflammation and enhance glucose-stimulated insulin secretion (GSIS) (54, 55). The possible antidiabetic activity mechanism of OJPs is summarized in Figure 4.

### 4.2 Cardioprotective activity

**Figure 5:**
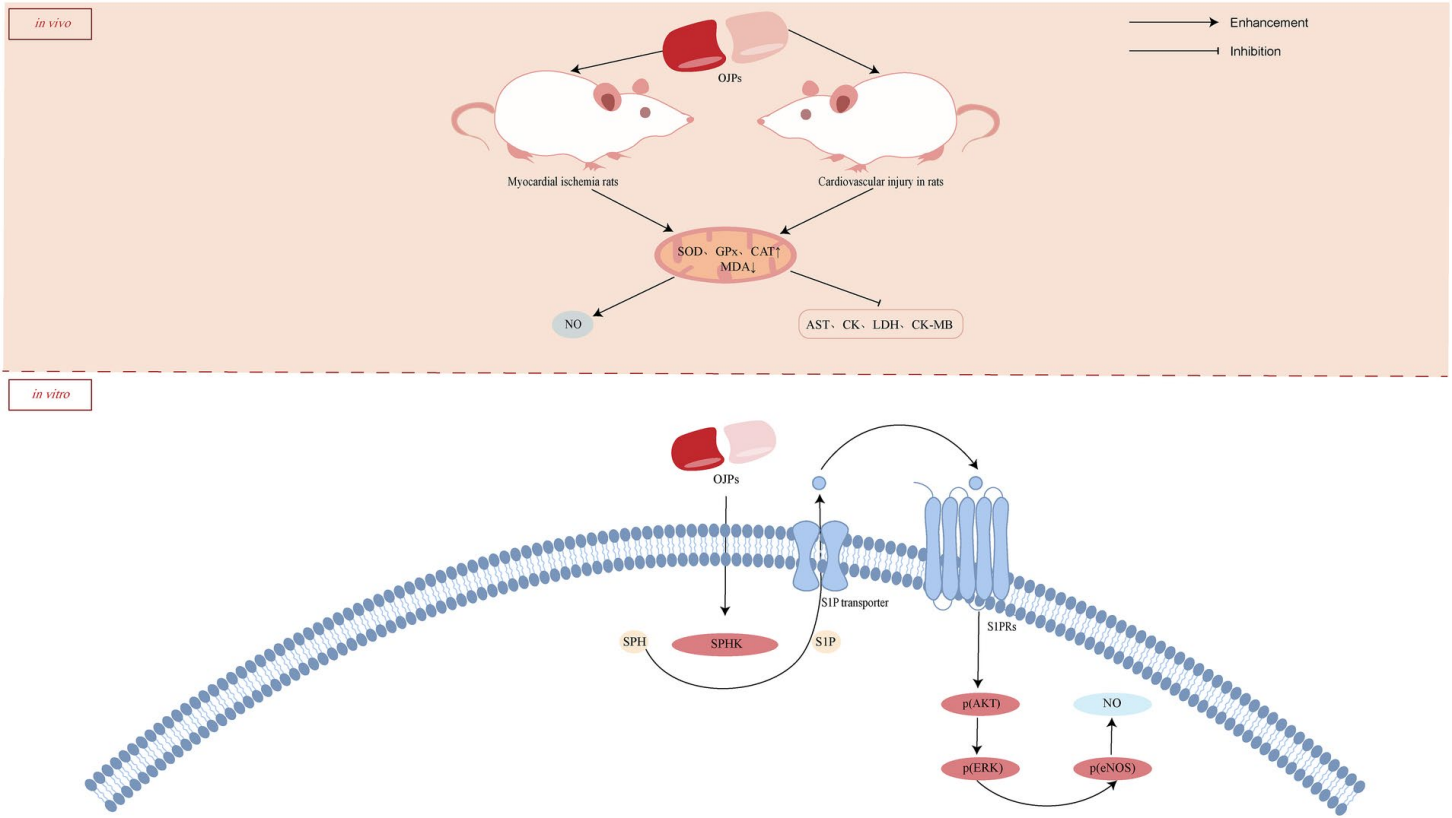
Schematic representation of the possible mechanism of cardioprotective activity of OJPs.

*Ophiopogon japonicus* has been widely used to treat cardiovascular diseases for thousands of years and has shown remarkable efficacy. In addition to clinical evidence, various experiments have confirmed that this drug has anti-arrhythmia, inhibition of platelet aggregation, reversal of myocardial ischemia, improvement of microcirculation and other effects (56, 57). As one of the main components of *O. japonicus*, OJP also showed cardioprotective effects. A water-soluble *β*-D-fructan (MDG-1) from *O. japonicus* protect cardiomyocytes and HMEC-1 cells from oxygen glucose deprivation (OGD)-induced injury and protect cardiomyocytes from ischemic death after coronary artery ligation in rats. Further mechanism studies showed that MDG-1 promoted the expression of sphingosine kinase 1 and sphingosine-1-phosphate (S1P) receptor 1, induced the expression of basic fibroblast growth factor (bFGF) in HMEC-1 cells, and induced Akt and extracellular signal-regulated kinase (ERK) phosphorylation in a dose-and time-dependent manner, thus protecting cardiomyocytes from ischemia-induced damage (58). S1P plays a crucial role in the occurrence and treatment of ischemic diseases. Wang et al. (59) also confirmed that exogenous MDG-1 stimulates the increase of S1P by activating SPHK, which leads to the phosphorylation of downstream signal effectors Akt and ERK through S1P receptor phosphorylation on the cell surface, thus exerting the effect of anti-myocardial ischemia. Oxidative stress causes cell death through multiple mechanisms and drives further damage of ischemic myocardium. In the rat model of isoproterenol (ISO)-induced myocardial injury, OJP (100, 200, and 300 mg/kg) could further improve myocardial enzyme indexes [aspartate aminotransferase (AST), lactate dehydrogenase (LDH), creatine kinase (CK) and creatine kinase isoenzyme-MB (CK-MB)] by reducing the elevation of ST segment and cardiac index, increasing the activities of superoxide dismutase (SOD), glutathione peroxidase (GPx) and catalase (CAT) in serum and myocardium, and reducing the level of malondialdehyde (MDA) (60). These results suggest that OJP protects cardiomyocytes from injury by enhancing endogenous antioxidant activity. Similarly, OJP increased antioxidant enzyme levels and improved cardiovascular function on cardiovascular injuries in diabetic rats (61). The possible cardioprotective activity mechanism of OJPs is summarized in Figure 5.

### 4.3 Immunomodulatory activity

Immune regulation is a physiological function of the body to recognize and exclude antigenic foreign substances to maintain physiological balance and stability. Abnormalities of the immune system can lead to a variety of diseases, such as rheumatoid arthritis, systemic lupus erythematosus, inflammatory bowel disease (62). Polysaccharides play an immunomodulatory role by activating immune cells (including T cells, B cells, dendritic cells, macrophages and natural killer cells), as well as by activating complement and promoting cytokine production (63).

Dendritic cells (DC) are the strongest antigen presenting cells at present, and they are the central link in initiating, regulating and maintaining immune response. Immature DC have strong migration ability and mature dendritic cells stimulate immune response by contacting T cells. OJPs promote the maturation of DC by prolonging the dendrites and increasing the level of nitric oxide (NO), thus improving the ability of immune response (64). Macrophages are not only the key participants of innate immune response, but also the important effector cells of immune response. There are mannose receptors, glucan receptors and polysaccharide receptors related to phagocytosis on the surface of activated macrophages. Phagocytosis of macrophages plays an important role in maintaining balance *in vivo* and is a key link of non-specific immunity (65, 66). *In vitro* and *in vivo* experiments showed that both OJP liposome (OPL) and OJP could significantly enhance the phagocytic activity of macrophages and play an immunomodulatory role (67, 68). Kupffer cells (KCs) is the largest number of macrophages in the body. Other experiments *in vitro* have found that OPL could significantly promote the phagocytosis of KCs, induce the secretion of nitric oxide (NO), induced nitric oxide synthase (iNOS), IL-6 and IL-12, and improve the expression of CD80 and CD86, thus enhance non-specific and specific immune function (69). Sulfonated modified OJP (OPS) enhanced its immune activity by promoting lymphocyte proliferation and increasing serum antibody titer, which was stronger than that of unmodified polysaccharides (70).

**Figure 6:**
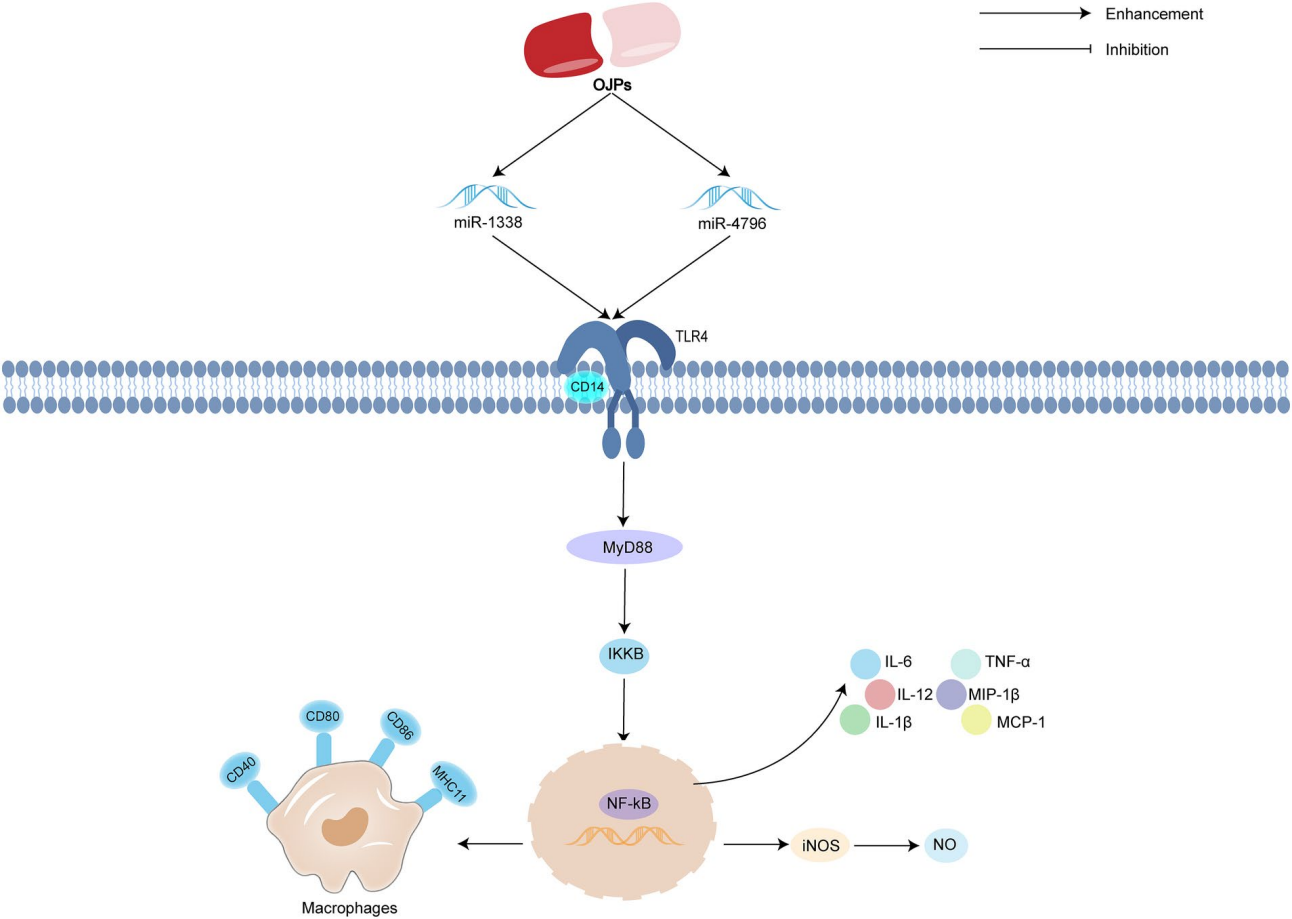
Schematic representation of the possible mechanism of immunomodulatory activity of OJPs.

MicroRNA (miRNA), as a small non-coding RNA molecule regulated by endogenous gene expression, binds to the target gene mRNA, regulates the expression of target genes at the post-transcriptional level, and then participates in pathophysiological processes such as development, immunity and metabolism (71). High-throughput sequencing confirmed that miR-1338 was related to the immunomodulatory activity of OJP. Further mechanism studies showed that after transfection of miR-1338 inhibitor, OJP promoted the secretion of NO, inducible iNOS and postoperative delirium (POD), improved phagocytosis and cell migration, increased the expression of mRNA and protein of toll-like receptor4 (TLR4), myeloid differentiation primary response gene 88 (MyD88), inhibitor of kappa B kinase (IKK *β*) and NF-κB, and inhibited cell apoptosis and ROS secretion. It is suggested that OJP increase the activity of KCs by regulating miR-1338 to activate TLR4-NF-κB signal pathway. OJP can increase the activity of macrophages by regulating miR-1338 to activate TLR4-NF-κB signal pathway (72). Cui et al. (73) also reached a similar conclusion that after transfection of miR-4796, OPL promote the secretion of NO, iNOS and reactive oxygen species (ROS), promote cell migration, phagocytosis and the expression of CD14 and major histocompatibility complex II (MHC II). The mRNA and protein expression levels of TLR4, MyD88 and NF-κB were significantly increased. These results suggest that OPL can regulate the immune activity of KCs by regulating miR-4796, activating TLR4-NF-κB signal pathway. The possible immunomodulatory activity mechanism of OJPs is summarized in Figure 6.

### 4.4 Regulating intestinal microbial and improving obesity activities

**Figure 7:**
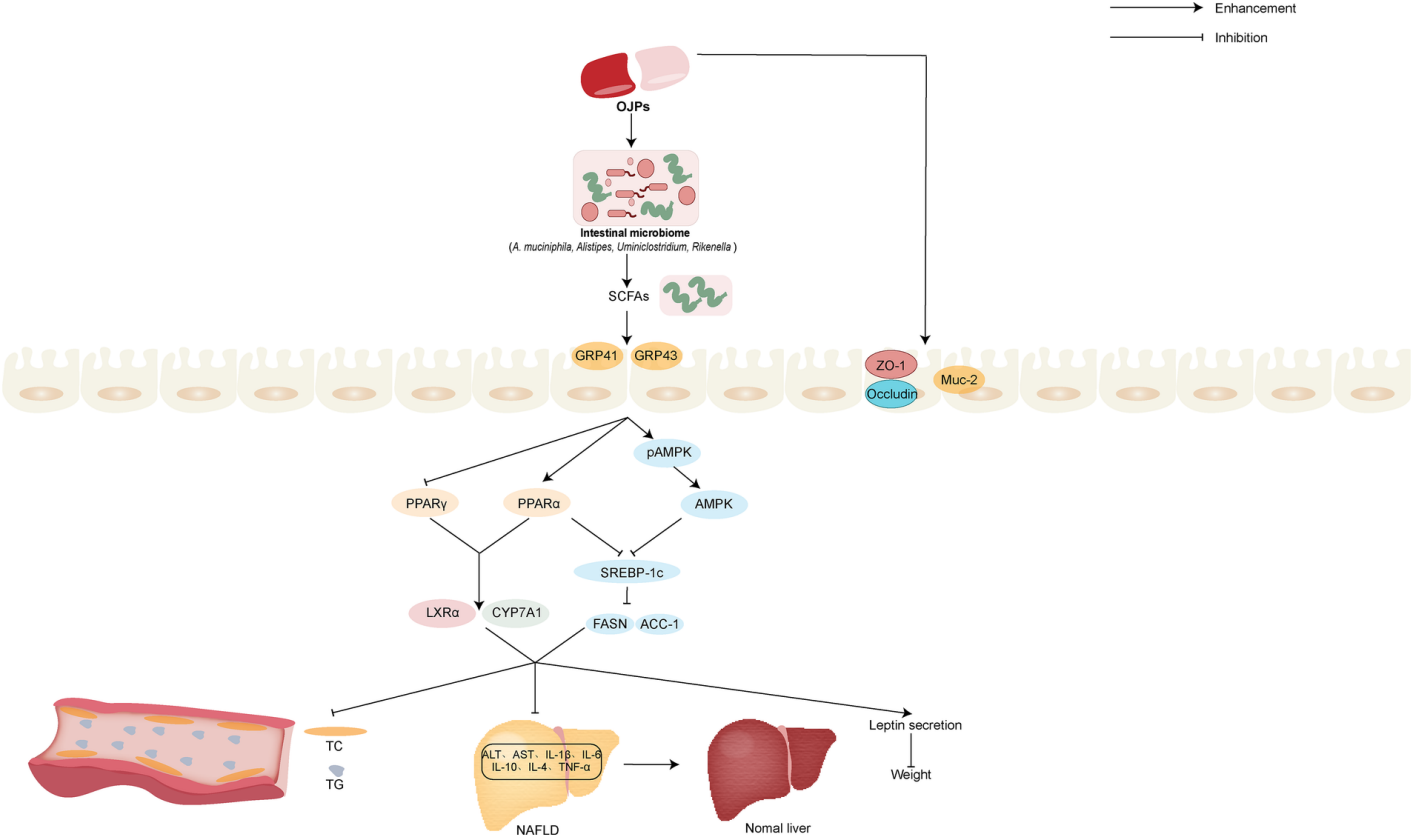
Schematic representation of the possible mechanism of OJPs involved in regulating gut microbial, improving obesity, hypolipidemic and hepatoprotective activity.

Due to sedentary lifestyles and excessive caloric intake, the prevalence of obesity is increasing globally each year. It is worth noting that obesity can cause a variety of diseases, including hypertension, dyslipidemia, type 2 diabetes, cardiovascular diseases, etc. (74). It is very necessary to seek safe and effective drugs to treat obesity and related diseases. MDG-1, a polysaccharide extracted from the roots of *O. japonicus*, can cause weight loss in obese mice without suppressing appetite or increasing physical activity, mainly by increasing oxygen and energy consumption, causing weight loss in obese animals and reducing the content of adipose tissue. In addition, MDG-1 can also improve plasma lipid profile, reduce leptin secretion, reduce liver lipid accumulation, and increase the expression of liver lipid and energy metabolism related genes (75). Zhang et al. (76) also confirmed that MDG-1 can control weight gain without inhibiting the appetite of mice fed a high-fat diet. Further study found that MDG-1 could increase the abundance of *A. muciniphila*. *A. muciniphila* stimulates the production of short chain fatty acids (SCFAs) to bind G-protein coupled receptor 41 (GPR41), G-protein coupled receptor 43 (GPR43) and adenosine 5′-monophosphate (AMP)-activated protein kinase (AMPK) and activate recipient PPARγ to limit energy intake and downregulate the glucose content. On the other hand, *A. muciniphila* depressed mRNA expression of SREBP-1c, FASN, ACC-1, PPARγ and increased PPAR *α* to inhibit lipogenesis and promote lipid differentiation and oxidation. Further restore lipid homeostasis in the host. Urinary metabolomics studies based on UPLC-TOF/MS have shown that MDG-1 can slightly improve metabolic disorders and weight gain in obese C57BL/6 mice (77). Gut microbiota, which refers to a complex ecosystem composed of bacteria, fungi, viruses and other microorganisms living in the human intestinal tract, has been gradually revealed in recent years in the mechanisms involved in host nutrition uptake, energy regulation and metabolic disorders (78, 79). The MDG-1 of 300 mg/kg was given to obese male C57BL/6 mice induced by high-fat diet for 12 weeks, the metabonomic analysis showed that MDG-1 could reduce the ratio of streptomyces to pseudomonas and regulate the abnormal gut microbiota to normal. In addition, MDG-1 can be degraded and utilized by intestinal microflora, which in turn can be absorbed and utilized by the host, thus playing a role in weight loss (80). The possible regulating intestinal microbial and improving obesity activity mechanism of OJPs is summarized in Figure 7.

### 4.5 Hypolipidemic and hepatoprotective activity

With the extensive development of antiviral therapy, the proportion of viral hepatitis caused by chronic hepatitis virus infection has decreased. The continuous improvement of people’s living standards and changes in lifestyle, compared with the past, people consume more high-calorie food, coupled with sedentary habits, which leads to the incidence of non-alcoholic fatty liver disease (NAFLD) is rising year by year. According to epidemiological data, the incidence of NAFLD in adults worldwide has reached 38% (81). Diabetic patients are often complicated with liver function injury and dyslipidemia, which affect the life span and quality of life of patients. *O. japonicus* polysaccharide (OJP1) had obvious reversal effect on triglyceride (TG), total cholesterol (TC), in diabetic rats, and biochemical and pathological analysis showed that OJP1 improved liver injury (14).

Gut microbiota and lipid metabolism have been proved to be closely related to the occurrence and development of NAFLD (82). In the model of NAFLD mice induced by high-fat diet, MDG-1 can improve the liver injury and reduce the accumulation of lipids in the liver. Further studies have shown that MDG-1 can increase the abundance of intestinal probiotics, such as *Alistipes*, *Ruminiclostridium*, and *Rikenella*, which are degraded into SCFAs by intestinal flora, regulate the level of AMPK signal pathway and lipid synthesis gene in liver, thus improve the lipid metabolism of HFD mice and reduce the process of NAFLD (45). *A. muciniphila* is a mucus-degrading bacteria in the intestinal tract, which produces SCFAs, activates G-protein-coupled cell surface receptors distributed in the intestine, liver and other tissues, increases energy intake and fat decomposition, and alleviates NAFLD (83). MDG-1 can promote SCFA-producing bacteria, including *A. muciniphila*, stimulate the production of SCFAs, thus improving liver lipogenesis and dyslipidemia caused by high-fat diet. Meanwhile, MDG-1 could improve intestinal mucosal proteins Occludin, Zonula occludens 1 (ZO-1) and recombinant mucin 2 (Muc-2), thus maintaining the permeability and integrity of intestinal barrier (76). Male C57BL/6 mice were randomly divided into three groups: low-fat diet, high-fat diet and high-fat diet plus 5% MDG-1, gene microarray analysis and reverse transcription-polymerase chain reaction (RT-PCR) showed that MDG-1 could inhibit PPARγ, activate PPAR*α*, up-regulate the expression of PPARs-related liver X receptor α (LXRα) and cholesterol 7-alpha hydroxylase (CYP7A1), and inhibit the level of sterol regulatory element-binding protein-1 (SREBP-1c) to accelerate cholesterol intake and excretion (84). The possible hypolipidemic and hepatoprotective activity mechanism of OJPs is summarized in Figure 7.

### 4.6 Antioxidant activity

Reactive oxygen species (ROS) are oxygen radicals found in living organisms, comprised of oxygen molecules and highly reactive oxygen-containing molecules (such as superoxide anion, hydroxyl radical, and hydrogen peroxide) (85). When the body is healthy, an adequate amount of ROS can eliminate aging and mutated cells. However, excessive ROS can lead to oxidative damage, degradation of proteins, deoxyribonucleic acid (DNA), and other biomolecules, resulting in aging, cancer, cardiovascular disease, diabetes, heart attacks, and other illnesses (86, 87). Hence, supplementing the body with antioxidants from external sources is essential to combat or alleviate oxidative stress. The potent antioxidant properties of natural polysaccharides have attracted increasing attention and interest from researchers (88, 89).

As a stable free radical, 2,2-diphenyl-1-picrylhydrazyl (DPPH) radical is easily scavenged by antioxidants, so free radical scavenging test is a common method to determine the antioxidant capacity of polysaccharides. Ultrasonic extraction of POJ-U1a steadily increased the scavenging activity of DPPH free radicals in the range of 0.25 ~ 8 mg/mL, and the scavenging ability of DPPH free radicals reached 35.08% at 8 mg/mL. Meanwhile, when the concentration of POJ-U1a was 8 mg/mL, the scavenging activity of hydroxyl radical and superoxide anion scavenging rate reached 32.50 and 45.01% respectively, which indicated that POJ-U1a had strong antioxidant activity (17). In clinical and experimental diabetes, the pathogenesis of diabetes has been proved to be closely related to oxidative stress. GPx, SOD and MDA can be used as indexes to evaluate the antioxidant activity of active substances (90). In the diabetic rat model, compared with the control group, OJP treatment significantly improved the levels of SOD, GPx and MDA in serum, liver and kidney of diabetic rats, which provided a theoretical basis for further study of OJP as an alternative means to prevent and reduce diabetes and its complications (14). In the H_2_O_2_-induced HaCaT cell injury or glucose-induced LO2 cell injury model, Fan et al. also confirmed that OJP can increase the SOD and NO production activity, and reduce the level of MDA to achieve antioxidant protection (11). Zhe MaiDong, Chuan MaiDong, Duanting Shan MaiDong, and Hubei MaiDong from different producing areas were obtained by the same extraction and purification method and named OJZP, OJCP, LMP and LSP, respectively. Further antioxidant capacity test showed that the antioxidant capacity of OJZP was similar to that of OJCP in scavenging 2,20-azino-bis (3-ethylbenzothiazoline-6-sulfonic acid) (ABTS) free radicals, which was much higher than that of LMP and LSP, indicating that OJP from different areas had different antioxidant capacity, which had a certain guiding significance for the cultivation and utilization of OJP (23).

### 4.7 Other activities

In addition to the above biological activities, OJPs also has other biological activities, such as anti-viral, anti-inflammatory, anti-apoptosis, renal protection, and anti-Sjogren’s syndrome activity. Wang et al. (91) found that *O. japonicus* polysaccharide (MDG-1) reduced glomerular mesangial dilatation and tubulointerstitial fibrosis and down-regulated the expression of transforming growth factor-beta 1 (TGF-*β*1) and connective tissue growth factor (CTGF) in diabetic nephropathy mice at 300 mg/kg, thus reducing extracellular matrix (ECM) deposition in renal tissue and achieving renal protective effect. 2,3,5,4′-tetrahydroxy stilbene-2-o-β-d-glucoside (THSG) is a polyhydroxy stilbene compound with a wide range of biological activities extracted from polygonum multiflorum. Due to its poor stability *in vitro*, poor intestinal absorption and short storage time in tissues, the application of THSG is limited. OJP could improve the water solubility and stability of THSG *in vitro* and prolong its pharmacological effect (92). In addition, OJP could promote the metabolism of Ophiopogon saponins and ginsenosides, which is mainly related to the stimulation of intestinal flora (33, 39). Li et al. (93) found that MDG-1 could reduce the proportion of Bax/Bcl-2 protein, the expression of caspase-3 and the secretion of inflammatory factors (TNF-*α*, IL-1β, IL-6, and Cox-2) in hydrogen peroxide (H_2_O_2_)-induced oxidative stress cell model of human umbilical vein endothelial cells (HUVECs) to protect HUVECs from H_2_O_2_-induced apoptosis and inflammation. In LPS-stimulated macrophage models, the levels of inflammatory factors NO and IL-8 were reduced after treatment with OJP (94, 95).

The value of cell A570 is an index reflecting the antiviral activity of polysaccharides and is positively correlated with the number of living cells. The average virus inhibition rate of unmodified *O. japonicus* polysaccharide (OPS) was 26.15%, and the antiviral activity was significantly enhanced after sulfation modification, up to 55.03% (27). As one of the common autoimmune diseases, Sjogren’s syndrome is mainly characterized by xerostomia and dry keratoconjunctivitis. In the mouse model of SS autoallergy, the decrease of saliva was improved, the amount of drinking water and SMG index increased after OJP treatment, but there was no regulation of food intake. Further analysis showed that OJP could decrease both the serum level of IFN-*γ* and IFN-γ/IL-4 ratio to regulate the Th1/Th2 cytokine imbalance (96).

## 5 Structural modification

The biological activity of polysaccharides mainly depends on its molecular structure. Structural modification (including physical, chemical and biological methods) can improve the biological activity of polysaccharides or make polysaccharides produce new activities (97, 98). Commonly used structural modifications include alkylation, acetylation, selenylation, carboxymethylation, and sulfonation.

### 5.1 Liposome modification

Liposome is a kind of artificial microvesicle whose water core is wrapped in one or more phospholipid bilayers. As an effective drug carrier, liposome can improve the efficacy and bioavailability of drugs. Compared with traditional polysaccharides, the combination of liposomes and polysaccharides not only has lower toxicity and fewer side effects, but also can enhance the biological activity of polysaccharides, such as anti-inflammation, antioxidation and immunomodulation, these properties can directly affect the physiological function of human body (99, 100). Usually, soy phospholipids and cholesterol are dissolved in chloroform, and then PBS containing OJP is injected into the chloroform solution. The mixture was homogenized to form a stable oil emulsion. Then the emulsion was evaporated into a colloid, hydrated with PBS, and then homogenized for 20 min to form a uniform liposome. Finally, OPL was obtained by filtering liposomes with 0.45 and 0.22 μm microporous membranes, respectively. Compared with OJP, OPL can significantly activate macrophages, promote the expression of IL-1β, TNF-*α*, monocyte chemoattractant protein 1 (MCP-1) and MIP-1β, and improve the level of immune response (68). *In vivo* experiments have also confirmed that compared with OJP, OPL could significantly improve the immune efficacy and reduce the mortality and morbidity of chicks, which may be due to the fact that OPL can enhance cellular and humoral immunity, and maintain higher lymphocyte proliferation, antibody titer and the proportion of CD4+ and CD8+ T lymphocyte subsets for a long time (67). In addition, OPL can increase the secretion of NO, iNOS and POD by regulating miR-1338, promote cell phagocytosis and migration, increase the expression of TLR4, MyD88, IKKβ and NF-κB mRNA and protein, inhibit cell apoptosis and ROS secretion, thereby improving Kupffer cell activity (72). In conclusion, the liposome of OJP can significantly enhance the immune-enhancing activity of OJP.

### 5.2 Polyethylene glycol modification

Polyethylene glycol (PEG) is a process in which PEG polymer chains are covalently attached to therapeutic molecules, which can significantly improve the pharmacokinetics of drugs, prolong the residence time *in vivo*, optimize tissue distribution and reduce adverse reactions (101). It is well known that OJP has anti-myocardial ischemia effect, but due to its short half-life *in vivo*, in order to improve the retention time of OJP in the body, using carboxymethylated radix ophiopogonis polysaccharide (ROP) and amino-terminated methoxy-PEG to form potentially bioactive PEGylated ROPs. Further study showed that compared to ROP (whose half-life is about 0.7 h), PEGylated ROPs have a half-life of 78.4 h in the blood after intravenous administration, and their mean residence time in the body is further significantly extended when given subcutaneously (102). Lin et al. (103) also confirmed that the half-life of PEGylated ROPs increased by 47.40 times and retained about 74% of ROP biological activity. The study on the tissue distribution of PEGylated ROP showed that the area under the curve of PEGylated ROP in the ischemic heart was about 1.60 times higher than that in the normal heart, indicating that polyethylene glycol could increase the myocardial plasma distribution of ROP and enhance the ability of anti-myocardial ischemia, which is helpful to the choice of cardiac targeted therapy strategy (104).

### 5.3 Sulfated modification

As a common and effective modification method, sulfonation modification can further improve the biological activity of polysaccharides. Sulfonation modification is mainly to replace the original hydroxyl groups of polysaccharides with sulfuric groups, so as to adjust the primary structure of the polysaccharide chain and reshape the spatial conformation of the polysaccharide chain (105). The main methods for sulfation of polysaccharides include sulfur trioxide-pyridine method, chlorosulfonic acid–pyridine (CSA–Pry) method, and concentrated sulfuric acid method (37). The modified conditions were as follows: the ratio of chlorosulfonic acid to pyridine was 1:4, the reaction temperature was 60°C, and the reaction time was 2 h to obtain sulfated Ophiopogonpolysaccharide3 (sOPS3). The virus inhibitory rates of sOPS3 (57.41%) were significantly higher than those in the unmodified OPS group (31.11%) (27). *O. japonicus* polysaccharide (OPS80 and OPSt) were sulfonated by CSA-Pry to obtain sOPS80 and sOPSt, *in vitro* experiments showed that the lymphocyte proliferation rate of OPS80 and OPSt groups was significantly higher than that of unmodified OPS group. *In vivo* experiments also confirmed that sulfated polysaccharides could increase the titer of serum antibodies and enhance their immune activity (70). Due to the existence of the-OSO3H group and the change of its spatial configuration, the sulfated OJP shows a higher ability to activate telomeric hydrocarbon and carbon atoms, thus giving it a stronger ability to donate hydrogen atoms and scavenge DPPH free radicals ability (15). Overall, sulfonation has proven to be an effective way to enhance the antiviral, antioxidant and immunomodulatory activities of OJPs.

## 6 Relationship between structure and biological activities

The relationship between structure and activity of polysaccharides is one of the main contents of glycobiology, and it is of great significance to clarify the relationship between structure and activity of polysaccharides. The biological activity of polysaccharides is related to their monomer composition, molecular weight, glycosidic bond type, molar ratio and chain skeleton. In order to find polysaccharides with good therapeutic and pharmacological effects, it is necessary to establish the relationship between the structure and activity of OJPs. The acquisition of OJPs is affected by many factors such as extraction, purification, and separation methods, resulting in poor repeatability. Therefore, based on existing research in the reference literature, we tried to summarize the structural factors that affect different pharmacological activities from multiple aspects such as monosaccharide composition, active functional groups and modification.

Xiong et al. (15) isolated four polysaccharide components from the tubers of *O. japonicus* by hot water extraction, which are OJP-1, OJP-2, OJP-3 and OJP-4. The content of caproic acid and sulfate showed that: OJP-4 > OJP-3 > OJP-2 > OJP-1. Further research found that OJP4 has a strong ability to scavenge DPPH free radicals and hydroxyl free radicals, which may be due to its stronger reducing and chelating ability on ferrous ions, indicating that caproic acid and sulfate are effective indicators of antioxidant activity of OJP. Differences in monosaccharide content also have different effects on the bioactivity of OJP. Pearson correlation analysis of pharmacological activity and physicochemical properties of OJP shows that Man content has the highest correlation with antioxidant capacity (0.90), followed by Xyl content (0.89). However, the most abundant monosaccharide Fru in OJP was significantly negatively correlated with antioxidant and immune activities (23). In addition, the biological activity of polysaccharides mainly depends on their molecular structure. Especially when some chemical groups are inserted, the flexibility and spatial structure of the polysaccharide chain change, which will enhance the activity of the polysaccharide or generate new activities. Sulfonation modification, as a commonly used chemical modification method, can significantly enhance the antiviral activity of OJP (27). Compared with OJP, OPL can significantly increase the phagocytic activity and IL-1, TNF-*α*, MCP-1, MIP-1 levels of mouse peritoneal macrophages, promote NO and iNOS secretion, and enhance the expression of CD14 and MHC-II co-stimulatory molecules (68).

In summary, current research on OJPs only focus on the relationship between local structure and activity, and there is no systematic study. Therefore, it is necessary to further identify the fine structure of OJPs and establish a multi-dimensional correlation with biological activities to clarify their structure–activity relationship.

## 7 Discussion

As a traditional Chinese herbal medicine with a long history of use, *O. japonicus* has attracted more and more attention. As its main active ingredient, OJPs have made certain progress in its extraction process. In addition to the traditional water extraction and alcohol precipitation methods, some scholars have also explored new extraction and separation technologies, such as ultrasound-assisted extraction, enzymatic extraction, microwave-assisted extraction, etc., these methods have high extraction rates and low consumption. However, compared with other component extraction and separation methods, OJPs extraction methods are relatively few and all have certain limitations. In future research, innovative methods can be explored to achieve higher extraction rates at lower costs and shorter processing times. The purification of OJPs mainly adopts gradient alcohol precipitation method and column chromatography. OJPs is mainly composed of Glc and Fru with molecular weight between 2.48 and 325 kDa. At present, most research on OJPs focuses on monosaccharide content and molecular weight, and further research is needed to explore their structural characteristics and glycosidic bonds. It has multiple biological activities such as hypoglycemic, cardioprotective, immunomodulatory, improvement of obesity, hepatoprotective, hypolipidemia, anti-oxidation, anti-tumor, anti-inflammation and renal protective activity. In addition, appropriate structural modifications can improve the pharmacological activity and bioavailability of OJPs. Currently, commonly used structural modifications of OJPs include liposome modification, sulfation modification, polyethylene glycol modification, etc. For example, liposome-modified OJP shows better immunoregulatory activity. Structural changes of OJPs can lead to changes in biological activities, and their pharmacological activities are related to monosaccharide composition, active functional groups and structural modifications.

Although a lot of progress has been made in the research of OJPs, there are still some problems that need to be solved. First of all, the current extraction methods of polysaccharides have been greatly developed, but no research has shown how different extraction techniques will affect the structural composition of OJPs. In addition, the current extraction, separation and purification schemes for OJPs are mainly limited to laboratories and may not be conducive to industrial scale. Select appropriate separation and purification methods based on the structural characteristics and research purposes of OJPs to improve the extraction rate and maximize the protection of the activity of OJPs. Second, the biological activity of polysaccharides is closely related to their structural characteristics. However, current structural research on OJPs focuses on molecular weight and monosaccharide composition. In the future, it is necessary to use more advanced analytical techniques such as x-ray diffraction, capillary electrophoresis and Raman spectroscopy to analyze the higher-order structure of OJPs, such as the composition of the primary structure of monosaccharides, the arrangement order of glycosyl groups, and the connection of glycosidic chains. Method, whether the sugar chain has branches, the size and position of the branches, etc. Additionally, as a biological molecule, OJPs have a high degree of diversity and complexity in its molecular structure. Currently, there is very little research on the structure and biological activity of OJPs. More research should focus on the relationship between the structure and biological activity of OJPs and the precise molecular mechanism behind it. Third, *in vivo* and *in vitro* experiments have confirmed various pharmacological activities of OJPs. However, the toxic and side effects and clinical pharmacological effects of OJPs have not been verified. Their pharmacological effects should be further explored in the future to lay the foundation for safe clinical application and expanded application.

Overall, with the deepening of research on OJPs, gratifying results have been achieved in their extraction process, structural characterization, pharmacological activities, modifications and applications, providing a comprehensive and profound foundation for their extensive development in the fields of medical care and functional food. Therefore, further research on its processing process, structure, biological activity and structure–activity relationship is indispensable.

## References

[1] Chen MH Leong F Gao SJ Chen X Lu JJ Lin LG. Comparison of Ophiopogon japonicus and *Liriope spicata* var. prolifera from different origins based on multi-component quantification and anticancer activity. Molecules. (2023) 28:1045. doi: 10.3390/molecules28031045, PMID: 36770712 PMC9920971

[2] Liu Q Lu JJ Hong HJ Yang Q Wang Y Chen XJ. Ophiopogon japonicus and its active compounds: a review of potential anticancer effects and underlying mechanisms. Phytomedicine. (2023) 113:154718. doi: 10.1016/j.phymed.2023.154718 36854203

[3] Fang J Wang X Lu M He X Yang X. Recent advances in polysaccharides from Ophiopogon japonicus and *Liriope spicata* var. prolifera. Int J Biol Macromol. (2018) 114:1257–66. doi: 10.1016/j.ijbiomac.2018.04.022, PMID: 29634971

[4] Wu W Ye K Wang ZH Guo L Zhu SS Wang Y. First report of *Meloidogyne arenaria* infecting Maidong (*Ophiopogon japonicus*) in China. Plant Dis. (2021) 105:4172. doi: 10.1094/PDIS-03-21-0584-PDN

[5] China Pharmacopoeia Commission, Chinese pharmacopeia, Beijing: China Medical Science Press. (2015) 26–155.

[6] Wu Z Zhao X Miyamoto A Zhao S Liu C Zheng W. Effects of steroidal saponins extract from *Ophiopogon japonicus* root ameliorates doxorubicin-induced chronic heart failure by inhibiting oxidative stress and inflammatory response. Pharm Biol. (2019) 57:176–83. doi: 10.1080/13880209.2019.1577467, PMID: 30860934 PMC6419681

[7] Zhao M Xu WF Shen HY Shen PQ Zhang J Wang DD. Comparison of bioactive components and pharmacological activities of ophiopogon japonicas extracts from different geographical origins. J Pharm Biomed Anal. (2017) 138:134–41. doi: 10.1016/j.jpba.2017.02.013, PMID: 28196345

[8] Yuan F Gao Q Tang H Shi J Zhou Y. Ophiopogonin-B targets PTP1B to inhibit the malignant progression of hepatocellular carcinoma by regulating the PI3K/AKT and AMPK signaling pathways. Mol Med Rep. (2022) 25:122. doi: 10.3892/mmr.2022.12638, PMID: 35169857 PMC8864608

[9] Zhan K Ji XL Luo L. Recent progress in research on *Momordica charantia* polysaccharides: extraction, purification, structural characteristics and bioactivities. Chem Biol Technol Agric. (2023) 10:58. doi: 10.1186/s40538-023-00433-4

[10] Fan Y Ma X Ma L Zhang J Zhang W Song X. Antioxidative and immunological activities of ophiopogon polysaccharide liposome from the root of *Ophiopogon japonicus*. Carbohydr Polym. (2016) 135:110–20. doi: 10.1016/j.carbpol.2015.08.089, PMID: 26453858

[11] Fan SR Wang JJ Mao YG Yuan J Jin LQ Chen XM. Characterization and antioxidant properties of OJP2, a polysaccharide isolated from *Ophiopogon japonicus*. Adv Biosci Biotechnol. (2015) 6:517–25. doi: 10.4236/abb.2015.68054

[12] Lei F Weckerle CS Heinrich M. Liriopogons (genera Ophiopogon and Liriope, Asparagaceae): a critical review of the phytochemical and pharmacological research. Front Pharmacol. (2021) 12:769929. doi: 10.3389/fphar.2021.769929, PMID: 34925027 PMC8678496

[13] Hou CY Yin MS Lan P Wang HR Nie H Ji XL. Recent progress in the research of Angelica sinensis (Oliv.) Diels polysaccharides: extraction, purification, structure and bioactivities. Chem Biol Technol Agric. (2021) 8:13. doi: 10.1186/s40538-021-00214-x

[14] Chen X Tang J Xie W Wang J Jin J Ren J. Protective effect of the polysaccharide from *Ophiopogon japonicus* on streptozotocin-induced diabetic rats. Carbohydr Polym. (2013) 94:378–85. doi: 10.1016/j.carbpol.2013.01.037, PMID: 23544552

[15] Xiong SL Li A Huang N Lu F Hou D. Antioxidant and immunoregulatory activity of different polysaccharide fractions from tuber of *Ophiopogon japonicus*. Carbohydr Polym. (2011) 86:1273–80. doi: 10.1016/j.carbpol.2011.06.025

[16] Ponmurugan K Al-Dhabi NA Maran JP Karthikeyan K Moothy IG Sivarajasekar N. Ultrasound assisted pectic polysaccharide extraction and its characterization from waste heads of Helianthus annus. Carbohydr Polym. (2017) 173:707–13. doi: 10.1016/j.carbpol.2017.06.018, PMID: 28732917

[17] Wang XM Sun RG Zhang J Chen YY Liu NN. Structure and antioxidant activity of polysaccharide POJ-U1a extracted by ultrasound from *Ophiopogon japonicus*. Fitoterapia. (2012) 83:1576–84. doi: 10.1016/j.fitote.2012.09.005, PMID: 22982331

[18] Borjigin G Wei F Jiang S Li Q Yang C. Extraction, purification, structural characterization and biological activity of polysaccharides from Fritillaria: a review. Int J Biol Macromol. (2023) 242:124817. doi: 10.1016/j.ijbiomac.2023.124817, PMID: 37182621

[19] Gu F Tao L Chen R Zhang J Wu X Yang M. Ultrasonic-cellulase synergistic extraction of crude polysaccharides from *Moringa oleifera* leaves and alleviation of insulin resistance in HepG2 cells. Int J Mol Sci. (2022) 23:12405. doi: 10.3390/ijms232012405, PMID: 36293262 PMC9604441

[20] Park JJ Lee WY. Anti-glycation effect of *Ecklonia cava* polysaccharides extracted by combined ultrasound and enzyme-assisted extraction. Int J Biol Macromol. (2021) 180:684–91. doi: 10.1016/j.ijbiomac.2021.03.118, PMID: 33766590

[21] Wang XY Zhang DD Yin JY Nie SP Xie MY. Recent developments in *Hericium erinaceus* polysaccharides: extraction, purification, structural characteristics and biological activities. Crit Rev Food Sci Nutr. (2019) 59:S96–S115. doi: 10.1080/10408398.2018.1521370 30421988

[22] Gu D Huang L Chen X Wu Q Ding K. Structural characterization of a galactan from Ophiopogon japonicus and anti-pancreatic cancer activity of its acetylated derivative. Int J Biol Macromol. (2018) 113:907–15. doi: 10.1016/j.ijbiomac.2018.03.019, PMID: 29518447

[23] Chen Z Zhu B Peng X Li S Zhao J. Quality evaluation of *Ophiopogon japonicus* from two authentic geographical origins in China based on physicochemical and pharmacological properties of their polysaccharides. Biomol Ther. (2022) 12:1491. doi: 10.3390/biom12101491, PMID: 36291700 PMC9599291

[24] Pan J Shi Y Zou J Zhang X Xin B Zhai B. Preparation technologies, structural features, and biological activities of polysaccharides from *Mesona chinensis* Benth.: a review. J Ethnopharmacol. (2024) 326:117979. doi: 10.1016/j.jep.2024.117979, PMID: 38412892

[25] Zhang Y Li L Ma X Liu R Shi R Zhao D. Extraction, purification, structural features, modifications, bioactivities, structure-activity relationships, and applications of polysaccharides from garlic: a review. Int J Biol Macromol. (2024) 265:131165. doi: 10.1016/j.ijbiomac.2024.131165, PMID: 38547941

[26] Chen X Jin J Tang J Wang ZF Wang JJ Jin LQ. Extraction, purification, characterization and hypoglycemic activity of a polysaccharide isolated from the root of *Ophiopogon japonicus*. Carbohydr Polym. (2011) 83:749–54. doi: 10.1016/j.carbpol.2010.08.050

[27] Zhang J Hu Y Wang D Qin T Liu C Liu X. The optimization of sulfation modification conditions for ophiopogon polysaccharide based on antiviral activity. Int J Biol Macromol. (2012) 51:657–62. doi: 10.1016/j.ijbiomac.2012.06.023, PMID: 22732131

[28] Wang FL Ji YB Yang B. Sulfated modification, characterization and monosaccharide composition analysis of *Undaria pinnatifida* polysaccharides and anti-tumor activity. Exp Ther Med. (2020) 20:630–6. doi: 10.3892/etm.2020.8720, PMID: 32537020 PMC7282060

[29] Sorokin AV Goncharova SS Lavlinskaya MS Holyavka MG Faizullin DA Zuev YF. Complexation of bromelain, Ficin, and papain with the graft copolymer of carboxymethyl cellulose sodium salt and N-Vinylimidazole enhances enzyme proteolytic activity. Int J Mol Sci. (2023) 24:11246. doi: 10.3390/ijms241411246, PMID: 37511006 PMC10379864

[30] Gong Y Zhang J Gao F Zhou J Xiang Z Zhou C. Structure features and in vitro hypoglycemic activities of polysaccharides from different species of Maidong. Carbohydr Polym. (2017) 173:215–22. doi: 10.1016/j.carbpol.2017.05.076, PMID: 28732860

[31] Li T Chen L Wu D Dong G Chen W Zhang H. The structural characteristics and biological activities of intracellular polysaccharide derived from mutagenic Sanghuangporus sanghuang strain. Molecules. (2020) 25:3693. doi: 10.3390/molecules25163693, PMID: 32823661 PMC7464456

[32] Zhang X Gao M Zhao X Qi Y Xu L Yin L. Purification and structural characterization of two polysaccharides with anti-inflammatory activities from *Plumbago zeylanica* L. Int J Biol Macromol. (2024) 260:129455. doi: 10.1016/j.ijbiomac.2024.129455, PMID: 38232876

[33] Wang HY Guo SC Peng ZT Wang C Duan R Dong TTX. Ophiopogon polysaccharide promotes the in vitro metabolism of Ophiopogonins by human gut microbiota. Molecules. (2019) 24:2886. doi: 10.3390/molecules24162886, PMID: 31398918 PMC6719028

[34] Fei Q Wang C Li Y Wang WM. The removal of polysaccharide pigment from *Ophiopogon japonicus* by activated carbon was investigated by single factor. Chin J Experiment Pharm. (2011) 17:48–50. doi: 10.3969/j.issn.1005-9903.2011.18.012

[35] Lin X Xu DS Feng Y Li SM Lu ZL Shen L. Release-controlling absorption enhancement of enterally administered *Ophiopogon japonicus* polysaccharide by sodium caprate in rats. J Pharm Sci. (2006) 95:2534–42. doi: 10.1002/jps.20738, PMID: 16937339

[36] Zhang J Wen C Gu J Ji C Duan Y Zhang H. Effects of subcritical water extraction microenvironment on the structure and biological activities of polysaccharides from Lentinus edodes. Int J Biol Macromol. (2019) 123:1002–11. doi: 10.1016/j.ijbiomac.2018.11.194, PMID: 30468806

[37] Guo J Lu L Li J Kang S Li G Li S. Extraction, structure, pharmacological activity, and structural modification of Lilium polysaccharides. Fitoterapia. (2024) 172:105760. doi: 10.1016/j.fitote.2023.105760, PMID: 38030097

[38] Wang HY Wang C Guo SC Chen ZC Peng ZT Duan R. Polysaccharide deriving from ophiopogonis Radix promotes metabolism of ginsenosides in the present of human gut microbiota based on UPLC-MS/MS assay. J Pharm Biomed Anal. (2019) 175:112779. doi: 10.1016/j.jpba.2019.112779, PMID: 31349212

[39] Rajendar B Reddy MVNJ Suresh CNV Rao GS Matur RV. O-phthalaldehyde based quantification of polysaccharide modification in conjugate vaccines. J Pharm Biomed Anal. (2024) 241:115995. doi: 10.1016/j.jpba.2024.115995, PMID: 38309096

[40] Liu M Zhang G Zhou K Wen J Zheng F Sun L. Structural characterization, antioxidant activity, and the effects of *Codonopsis pilosula* polysaccharides on the solubility and stability of flavonoids. J Pharm Biomed Anal. (2023) 229:115368. doi: 10.1016/j.jpba.2023.115368, PMID: 37001273

[41] Zhu B Zhang W Zhao J Chen B Liu F Li S. Characterization and comparison of bioactive polysaccharides from *Grifola frondosa* by HPSEC-MALLS-RID and saccharide mapping based on HPAEC-PAD. Polymers. (2022) 15:208. doi: 10.3390/polym15010208, PMID: 36616557 PMC9824690

[42] Hu C Li HX Zhang MT Liu LF. Structure characterization and anticoagulant activity of a novel polysaccharide from *Leonurus artemisia* (Laur.) S. Y. Hu F. RSC Adv. (2020) 10:2254–66. doi: 10.1039/c9ra10853j, PMID: 35494573 PMC9048717

[43] Shu L Lin S Zhou S Yuan T. Glycan-lectin interactions between platelets and tumor cells drive hematogenous metastasis. Platelets. (2024) 35:2315037. doi: 10.1080/09537104.2024.2315037, PMID: 38372252

[44] Ji XL Cheng YQ Tian J Zhang SQ Jing YS Shi MM. Structural characterization of polysaccharide from jujube (*Ziziphus jujuba* mill.) fruit. Chem Biol Technol Agric. (2021) 8:54. doi: 10.1186/s40538-021-00255-2

[45] Wang X Shi L Wang X Feng Y Wang Y. MDG-1, an Ophiopogon polysaccharide, restrains process of non-alcoholic fatty liver disease via modulating the gut-liver axis. Int J Biol Macromol. (2019) 141:1013–21. doi: 10.1016/j.ijbiomac.2019.09.007, PMID: 31491513

[46] Wang J An G Peng X Zhong F Zhao K Qi L. Effects of three Huanglian-derived polysaccharides on the gut microbiome and fecal metabolome of high-fat diet/streptozocin-induced type 2 diabetes mice. Int J Biol Macromol. (2024) 273:133060. doi: 10.1016/j.ijbiomac.2024.133060, PMID: 38871107

[47] Ji XL Guo JH Cao TZ Zhang TT Liu YQ Yan YZ. Review on mechanisms and structure-activity relationship of hypoglycemic effects of polysaccharides from natural resources. Food Sci Human Wellness. (2023) 12:1969–80. doi: 10.1016/j.fshw.2023.03.017

[48] Hennebelle A Ismail L Materwala H Al Kaabi J Ranjan P Janardhanan R. Secure and privacy-preserving automated machine learning operations into end-to-end integrated IoT-edge-artificial intelligence-blockchain monitoring system for diabetes mellitus prediction. Comput Struct Biotechnol J. (2023) 23:212–33. doi: 10.1016/j.csbj.2023.11.038, PMID: 38169966 PMC10758733

[49] Fu H You S Zhao D An Q Zhang J Wang C. Tremella fuciformis polysaccharides inhibit UVA-induced photodamage of human dermal fibroblast cells by activating up-regulating Nrf2/Keap1 pathways. J Cosmet Dermatol. (2021) 20:4052–9. doi: 10.1111/jocd.14051, PMID: 33686752

[50] Wang LY Wang Y Xu DS Ruan KF Feng Y Wang S. MDG-1, a polysaccharide from *Ophiopogon japonicus* exerts hypoglycemic effects through the PI3K/Akt pathway in a diabetic KKAy mouse model. J Ethnopharmacol. (2012) 143:347–54. doi: 10.1016/j.jep.2012.06.050, PMID: 22776833

[51] Hafiane A. Adiponectin-mediated regulation of the adiponectin cascade in cardiovascular disease: updates. Biochem Biophys Res Commun. (2024) 694:149406. doi: 10.1016/j.bbrc.2023.149406, PMID: 38134479

[52] Wang H. Preventive effects of ophiopogon-polysaccharide on apiponectin in gestational diabetes mellitus rat. Asian Pac J Trop Med. (2013) 6:296–9. doi: 10.1016/S1995-7645(13)60059-0, PMID: 23608331

[53] Xu J Wang Y Xu DS Ruan KF Feng Y Wang S. Hypoglycemic effects of MDG-1, a polysaccharide derived from Ophiopogon japonicas, in the Ob/Ob mouse model of type 2 diabetes mellitus. Int J Biol Macromol. (2011) 49:657–62. doi: 10.1016/j.ijbiomac.2011.06.026, PMID: 21756932

[54] Mao D Tian XY Mao D Hung SW Wang CC Lau CBS. A polysaccharide extract from the medicinal plant Maidong inhibits the IKK-NF-κB pathway and IL-1β-induced islet inflammation and increases insulin secretion. J Biol Chem. (2020) 295:12573–87. doi: 10.1074/jbc.RA120.014357, PMID: 32605924 PMC7476719

[55] Zhu Y Cong W Shen L Wei H Wang Y Wang L. Fecal metabonomic study of a polysaccharide, MDG-1 from *Ophiopogon japonicus* on diabetic mice based on gas chromatography/time-of-flight mass spectrometry (GC TOF/MS). Mol BioSyst. (2014) 10:304–12. doi: 10.1039/c3mb70392d, PMID: 24292023

[56] He F Xu BL Chen C Jia HJ Wu JX Wang XC. Methylophiopogonanone a suppresses ischemia/reperfusion-induced myocardial apoptosis in mice via activating PI3K/Akt/eNOS signaling pathway. Acta Pharmacol Sin. (2016) 37:763–71. doi: 10.1038/aps.2016.14, PMID: 27063216 PMC4954762

[57] Kou J Tian Y Tang Y Yan J Yu B. Antithrombotic activities of aqueous extract from Radix Ophiopogon japonicus and its two constituents. Biol Pharm Bull. (2006) 29:1267–70. doi: 10.1248/bpb.29.1267, PMID: 16755031

[58] Wang S Zhang Z Lin X Xu DS Feng Y Ding K. A polysaccharide, MDG-1, induces S1P1 and bFGF expression and augments survival and angiogenesis in the ischemic heart. Glycobiology. (2010) 20:473–84. doi: 10.1093/glycob/cwp199, PMID: 20008963

[59] Wang S Lin X Wang LY Ruan KF Feng Y Li XY. A polysaccharides MDG-1 augments survival in the ischemic heart by inducing S1P release and S1P1 expression. Int J Biol Macromol. (2012) 50:734–40. doi: 10.1016/j.ijbiomac.2011.12.005, PMID: 22197795

[60] Fan S Zhang J Xiao Q Liu P Zhang Y Yao E. Cardioprotective effect of the polysaccharide from *Ophiopogon japonicus* on isoproterenol-induced myocardial ischemia in rats. Int J Biol Macromol. (2020) 147:233–40. doi: 10.1016/j.ijbiomac.2020.01.068, PMID: 31923517

[61] Zhang J Fan S Mao Y Ji Y Jin L Lu J. Cardiovascular protective effect of polysaccharide from *Ophiopogon japonicus* in diabetic rats. Int J Biol Macromol. (2016) 82:505–13. doi: 10.1016/j.ijbiomac.2015.09.069, PMID: 26434529

[62] Majdalawieh AF Yousef SM Abu-Yousef IA Nasrallah GK. Immunomodulatory and anti-inflammatory effects of sesamin: mechanisms of action and future directions. Crit Rev Food Sci Nutr. (2022) 62:5081–112. doi: 10.1080/10408398.2021.1881438, PMID: 33544009

[63] Liu Z Liao L Chen Q Lin S Luo Y Qin T. Effects of Hericium erinaceus polysaccharide on immunity and apoptosis of the main immune organs in Muscovy duck reovirus-infected ducklings. Int J Biol Macromol. (2021) 171:448–56. doi: 10.1016/j.ijbiomac.2020.12.222, PMID: 33421472

[64] Zhu N Lv X Wang Y Li J Liu Y Lu W. Comparison of immunoregulatory effects of polysaccharides from three natural herbs and cellular uptake in dendritic cells. Int J Biol Macromol. (2016) 93:940–51. doi: 10.1016/j.ijbiomac.2016.09.064, PMID: 27659001

[65] Cai W Lian L Li A Zhang Q Li M Zhang J. Cardiac resident macrophages: the core of cardiac immune homeostasis. Cell Signal. (2024) 119:111169. doi: 10.1016/j.cellsig.2024.111169, PMID: 38599440

[66] Joosten SA Ottenhoff TH. Human CD4 and CD8 regulatory T cells in infectious diseases and vaccination. Hum Immunol. (2008) 69:760–70. doi: 10.1016/j.humimm.2008.07.017 18835413

[67] Fan Y Ma X Zhang J Ma L Gao Y Zhang W. Ophiopogon polysaccharide liposome can enhance the non-specific and specific immune response in chickens. Carbohydr Polym. (2015) 119:219–27. doi: 10.1016/j.carbpol.2014.11.048, PMID: 25563963

[68] Sun W Hu W Meng K Yang L Zhang W Song X. Activation of macrophages by the ophiopogon polysaccharide liposome from the root tuber of *Ophiopogon japonicus*. Int J Biol Macromol. (2016) 91:918–25. doi: 10.1016/j.ijbiomac.2016.06.037, PMID: 27311507

[69] Fan Y Song X Gao Y Chen Y Ma L Zhang W. Preparation and optimization of ophiopogon polysaccharide liposome and its activity on Kupffer cells. Int J Pharm. (2014) 477:421–30. doi: 10.1016/j.ijpharm.2014.10.053, PMID: 25445522

[70] Zhang J Chen J Wang D Hu Y Zhang C Qin T. Immune-enhancing activity comparison of sulfated ophiopogon polysaccharide and sulfated jujube polysaccharide. Int J Biol Macromol. (2013) 52:212–7. doi: 10.1016/j.ijbiomac.2012.09.025, PMID: 23041668

[71] Vo TH El-Sherbieny Abdelaal E Jordan E O'Donovan O McNeela EA Mehta JP. miRNAs as biomarkers of therapeutic response to HER2-targeted treatment in breast cancer: a systematic review. Biochem Biophys Rep. (2023) 37:101588. doi: 10.1016/j.bbrep.2023.101588, PMID: 38088952 PMC10711031

[72] Duan X Pan X Cui J Ke L Liu J Song X. The effect of miR-1338 on the immunomodulatory activity of ophiopogon polysaccharide liposome. Int J Biol Macromol. (2021) 193:1871–84. doi: 10.1016/j.ijbiomac.2021.11.019, PMID: 34774589

[73] Cui J Pan X Duan X Ke L Song X Zhang W. Ophiopogon polysaccharide liposome regulated the immune activity of Kupffer cell through miR-4796. Int J Mol Sci. (2022) 23:14659. doi: 10.3390/ijms232314659, PMID: 36498983 PMC9735683

[74] Orsso CE Vieira FT Basuray N Duke RL Pakseresht M Rubin DA. The metabolic load-capacity model and cardiometabolic health in children and youth with obesity. Pediatr Obes. (2024) 19:e13098. doi: 10.1111/ijpo.13098, PMID: 38263541

[75] Wang Y Zhu Y Ruan K Wei H Feng Y. MDG-1, a polysaccharide from *Ophiopogon japonicus*, prevents high fat diet-induced obesity and increases energy expenditure in mice. Carbohydr Polym. (2014) 114:183–9. doi: 10.1016/j.carbpol.2014.08.013, PMID: 25263880

[76] Zhang L Wang Y Wu F Wang X Feng Y Wang Y. MDG, an *Ophiopogon japonicus* polysaccharide, inhibits non-alcoholic fatty liver disease by regulating the abundance of *Akkermansia muciniphila*. Int J Biol Macromol. (2022) 196:23–34. doi: 10.1016/j.ijbiomac.2021.12.036, PMID: 34920070

[77] Li Y Zhu YY Shi LL Shen L Wei H Wang Y. UPLC-TOF/MS based urinary metabonomic studies reveal mild prevention effects of MDG-1 on metabolic disorders in diet-induced obese mice. Anal Methods. (2014) 6:4171–80. doi: 10.1039/C4AY00796D

[78] Ji X Hou C Gao Y Xue Y Yan Y Guo X. Metagenomic analysis of gut microbiota modulatory effects of jujube (*Ziziphus jujuba* mill.) polysaccharides in a colorectal cancer mouse model. Food Funct. (2020) 11:163–73. doi: 10.1039/c9fo02171j, PMID: 31830158

[79] Qi L Wu S Liu N Zhang X Li P Xia L. *Salvia miltiorrhiza* bunge extract improves the Th17/Treg imbalance and modulates gut microbiota of hypertensive rats induced by high-salt diet. J Funct Foods. (2024) 117:106211. doi: 10.1016/j.jff.2024.106211

[80] Shi LL Li Y Wang Y Feng Y. MDG-1, an Ophiopogon polysaccharide, regulate gut microbiota in high-fat diet-induced obese C57BL/6 mice. Int J Biol Macromol. (2015) 81:576–83. doi: 10.1016/j.ijbiomac.2015.08.057, PMID: 26321425

[81] Golabi P Owrangi S Younossi ZM. Global perspective on non-alcoholic fatty liver disease and non-alcoholic steatohepatitis-prevalence, clinical impact, economic implications and management strategies. Aliment Pharmacol Ther. (2024) 59 Suppl 1:S1–9. doi: 10.1111/apt.17833, PMID: 38813821

[82] Chu H Duan Y Yang L Schnabl B. Small metabolites, possible big changes: a microbiota-centered view of non-alcoholic fatty liver disease. Gut. (2019) 68:359–70. doi: 10.1136/gutjnl-2018-316307, PMID: 30171065

[83] Sivaprakasam S Prasad PD Singh N. Benefits of short-chain fatty acids and their receptors in inflammation and carcinogenesis. Pharmacol Ther. (2016) 164:144–51. doi: 10.1016/j.pharmthera.2016.04.007, PMID: 27113407 PMC4942363

[84] Wang X Shi L Joyce S Wang Y Feng Y. MDG-1, a potential regulator of PPARα and PPARγ, ameliorates dyslipidemia in mice. Int J Mol Sci. (2017) 18:1930. doi: 10.3390/ijms18091930, PMID: 28885549 PMC5618579

[85] Ji XL Guo JH Ding DQ Gao J Hao LR Guo XD. Structural characterization and antioxidant activity of a novel high-molecular-weight polysaccharide from *Ziziphus Jujuba* cv. Muzao Food Measure. (2022) 16:2191–200. doi: 10.1007/s11694-022-01288-3

[86] Koletti A Skliros D Kalloniati C Marka S Zografaki ME Infante C. Global omics study of Tetraselmis chuii reveals time-related metabolic adaptations upon oxidative stress. Appl Microbiol Biotechnol. (2024) 108:138. doi: 10.1007/s00253-023-12936-z, PMID: 38229403 PMC10791844

[87] Qin D Han S Liu M Guo T Hu Z Zhou Y. Polysaccharides from Phellinus linteus: a systematic review of their extractions, purifications, structures and functions. Int J Biol Macromol. (2023) 230:123163. doi: 10.1016/j.ijbiomac.2023.123163, PMID: 36623622

[88] Wu Y Liu H Li Z Huang D Nong L Ning Z. Purification of polysaccharides from Phellinus linteus by using an aqueous two-phase system and evaluation of the physicochemical and antioxidant properties of polysaccharides in vitro. Prep Biochem Biotechnol. (2022) 52:89–98. doi: 10.1080/10826068.2021.1911815, PMID: 33939578

[89] Mu S Yang W Huang G. Antioxidant activities and mechanisms of polysaccharides. Chem Biol Drug Des. (2021) 97:628–32. doi: 10.1111/cbdd.13798 32946177

[90] Algefare AI Alfwuaires M Famurewa AC Elsawy H Sedky A. Geraniol prevents CCl4-induced hepatotoxicity via suppression of hepatic oxidative stress, pro-inflammation and apoptosis in rats. Toxicol Rep. (2024) 12:128–34. doi: 10.1016/j.toxrep.2024.01.007, PMID: 38304701 PMC10831491

[91] Wang Y Shi LL Wang LY Xu JW Feng Y. Protective effects of MDG-1, a polysaccharide from *Ophiopogon japonicus* on diabetic nephropathy in diabetic KKAy mice. Int J Mol Sci. (2015) 16:22473–84. doi: 10.3390/ijms160922473, PMID: 26393572 PMC4613319

[92] Sun LL Wang M Zhang HJ You GJ Liu YN Ren XL. The influence of polysaccharides from *Ophiopogon japonicus* on 2,3,5,4′-tetrahydroxy-stilbene-2-O-β-d-glucoside about biopharmaceutical properties in vitro and pharmacokinetics in vivo. Int J Biol Macromol. (2018) 119:677–82. doi: 10.1016/j.ijbiomac.2018.07.179, PMID: 30067956

[93] Li LC Wang ZW Hu XP Wu ZY Hu ZP Ruan YL. MDG-1 inhibits H2O2-induced apoptosis and inflammation in human umbilical vein endothelial cells. Mol Med Rep. (2017) 16:3673–9. doi: 10.3892/mmr.2017.6957, PMID: 28713956

[94] Lin C Hsu FY Lin WT Cha CY Ho YC Mi FL. Biodegradable nanoparticles prepared from chitosan and casein for delivery of bioactive polysaccharides. Polymers. (2022) 14:2966. doi: 10.3390/polym14142966, PMID: 35890742 PMC9315736

[95] Lin C Kuo TC Lin JC Ho YC Mi FL. Delivery of polysaccharides from *Ophiopogon japonicus* (OJPs) using OJPs/chitosan/whey protein co-assembled nanoparticles to treat defective intestinal epithelial tight junction barrier. Int J Biol Macromol. (2020) 160:558–70. doi: 10.1016/j.ijbiomac.2020.05.151, PMID: 32464213

[96] Wang Y Yan T Shen J Guo H Xiang X. Preventive effect of *Ophiopogon japonicus* polysaccharides on an autoallergic mouse model for Sjogren's syndrome by regulating the Th1/Th2 cytokine imbalance. J Ethnopharmacol. (2007) 114:246–53. doi: 10.1016/j.jep.2007.08.014, PMID: 17888600

[97] Jiao J Yu J Ji H Liu A. Synthesis of macromolecular Astragalus polysaccharide-nano selenium complex and the inhibitory effects on HepG2 cells. Int J Biol Macromol. (2022) 211:481–9. doi: 10.1016/j.ijbiomac.2022.05.095, PMID: 35584715

[98] Zhang B Lan W Wang Z Shao Z Xie J. Modified chitosan with different phenolic acids: characterization, physicochemical properties, and biological activity. Food Chem. (2024) 441:138337. doi: 10.1016/j.foodchem.2023.138337, PMID: 38199114

[99] Li Y Jia G Li T Zhou X Zhao H Cao J. Preparation optimization and immunological activity studies of *Portulaca oleracea* L. polysaccharides liposomes. Curr Pharm Des. (2024) 30:786–97. doi: 10.2174/0113816128279071231204071210, PMID: 38385493

[100] Liu D Hou T Geng C Song L Hou X Chen Y. Liposomes enhance the immunological activity of Polygonatum Cyrtonema Hua polysaccharides. J Pharm Sci. (2024) 113:1572–9. doi: 10.1016/j.xphs.2024.01.005, PMID: 38237668

[101] Gong L Zhu J Yang Y Qiao S Ma L Wang H. Effect of polyethylene glycol on polysaccharides: from molecular modification, composite matrixes, synergetic properties to embeddable application in food fields. Carbohydr Polym. (2024) 327:121647. doi: 10.1016/j.carbpol.2023.121647, PMID: 38171672

[102] Lin X Wang ZJ Huang F Liang S Shen L Feng Y. Long-circulating delivery of bioactive polysaccharide from radix ophiopogonis by PEGylation. Int J Nanomedicine. (2011) 6:2865–72. doi: 10.2147/IJN.S26306, PMID: 22131832 PMC3224713

[103] Lin X Wang S Jiang Y Wang ZJ Sun GL Xu DS. Poly (ethylene glycol)-radix Ophiopogonis polysaccharide conjugates: preparation, characterization, pharmacokinetics and in vitro bioactivity. Eur J Pharm Biopharm. (2010) 76:230–7. doi: 10.1016/j.ejpb.2010.07.003, PMID: 20633648

[104] Lin X Wang ZJ Wang S Shen L Feng Y Ruan KF. Comparison of tissue distribution of a PEGylated Radix Ophiopogonis polysaccharide in mice with normal and ischemic myocardium. Eur J Pharm Biopharm. (2011) 79:621–6. doi: 10.1016/j.ejpb.2011.07.002, PMID: 21784151

[105] Li Z Sang R Feng G Feng Y Zhang R Yan X. Microbiological and metabolic pathways analysing the mechanisms of alfalfa polysaccharide and sulfated alfalfa polysaccharide in alleviating obesity. Int J Biol Macromol. (2024) 263:130334. doi: 10.1016/j.ijbiomac.2024.130334, PMID: 38387635

